# Two-dimensional carbon-based nanocomposites for photocatalytic energy generation and environmental remediation applications

**DOI:** 10.3762/bjnano.8.159

**Published:** 2017-08-03

**Authors:** Suneel Kumar, Ashish Kumar, Ashish Bahuguna, Vipul Sharma, Venkata Krishnan

**Affiliations:** 1 School of Basic Sciences and Advanced Materials Research Center, Indian Institute of Technology Mandi, Kamand, Mandi 175005, H.P., India

**Keywords:** energy generation, environmental remediation, graphene, graphitic carbon nitride, nanocomposites, photocatalysis, two-dimensional carbon

## Abstract

In the pursuit towards the use of sunlight as a sustainable source for energy generation and environmental remediation, photocatalytic water splitting and photocatalytic pollutant degradation have recently gained significant importance. Research in this field is aimed at solving the global energy crisis and environmental issues in an ecologically-friendly way by using two of the most abundant natural resources, namely sunlight and water. Over the past few years, carbon-based nanocomposites, particularly graphene and graphitic carbon nitride, have attracted much attention as interesting materials in this field. Due to their unique chemical and physical properties, carbon-based nanocomposites have made a substantial contribution towards the generation of clean, renewable and viable forms of energy from light-based water splitting and pollutant removal. This review article provides a comprehensive overview of the recent research progress in the field of energy generation and environmental remediation using two-dimensional carbon-based nanocomposites. It begins with a brief introduction to the field, basic principles of photocatalytic water splitting for energy generation and environmental remediation, followed by the properties of carbon-based nanocomposites. Then, the development of various graphene-based nanocomposites for the above-mentioned applications is presented, wherein graphene plays different roles, including electron acceptor/transporter, cocatalyst, photocatalyst and photosensitizer. Subsequently, the development of different graphitic carbon nitride-based nanocomposites as photocatalysts for energy and environmental applications is discussed in detail. This review concludes by highlighting the advantages and challenges involved in the use of two-dimensional carbon-based nanocomposites for photocatalysis. Finally, the future perspectives of research in this field are also briefly mentioned.

## Review

### Introduction

The problems of global energy shortage and environmental pollution are continuously increasing and various research groups are working to develop an alternative for the depleting fossil fuel reserves to effectively address the energy crisis and other environmental issues [1–2]. Moreover, the immense industrialization and rapid population increase has generated more demand for clean water sources all over the world. This demand has been continuously increasing due to the inevitable discharge of pollutants into the natural water cycle from various pharmaceutical and food industries [3]. Hence, there is an urgent need to develop green (ecologically-friendly), sustainable and technologically promising approaches to generate clean energy as well as to completely degrade pollutants into CO_2_ and H_2_O. Hydrogen seems to be a promising solution as a sustainable, clean and renewable energy source to overcome this energy crisis [4]. Hydrogen is mainly present in fossil fuels, such as natural gas and coal, from which it can be produced through steam reforming, partial oxidation, coal gasification and other processes [4]. However, these methods are mainly restricted due to carbon dioxide emission into the environment and high costs [4–5]. As hydrogen is an abundant element and present in nature in the form of water, its production from water using solar energy is therefore an area of immense interest for researchers because of its potential to fulfil the global energy demand and related environmental issues [5].

For the first time, photoelectrochemical (PEC) hydrogen production was achieved in 1972 by Fujishima and Honda on a TiO_2_ anode and Pt cathode under ultraviolet (UV) light irradiation [6]. After this, research interest in exploring semiconductors for hydrogen production has grown significantly and many research groups have focussed their studies in this direction [7–10]. Hence, in the recent decade, heterogeneous photocatalysis has been widely explored for the conversion of solar energy into chemical energy and for pollutant removal from water [11–12]. Up to now, various interesting semiconductors such as TiO_2_, ZnO, WO_3_, CdS, Bi_2_O_3_, Fe_2_O_3_, SnO_2_, BiVO_4_, etc. have been investigated for hydrogen evolution reactions and environmental remediation applications [13–19].

In the last 25 years, the emergence of carbon-based nanomaterials has opened new ways of harvesting solar energy and generation of clean energy in the form of hydrogen [20–21]. Carbon is one of the most abundant elements on the earth. In the past two decades, carbon-based materials such as graphene, graphitic carbon nitride (g-C_3_N_4_), fullerenes and carbon nanotubes (CNTs) have been explored for various applications such as Li-ion batteries [22], supercapacitors [23], energy storage [24], biosensors [25], molecular imaging [26], fuel cells [27] and catalysis [28]. The non-toxicity, abundance and the environmentally benign nature of these carbon-based materials makes them a remarkable class of materials with unique electrical and optical properties for diverse applications.

In recent times, carbon-based materials and semiconductor nanocomposites have attracted great attention and significant progress has been achieved in the field of photocatalysis. In this regard, much of the pioneering work on nanocarbon–semiconductor interface engineering has been reported by D. Eder and M. Prato for environmental remediation and energy generation applications [29–32]. Semiconductor nanocomposite-based photocatalytic reactions are generally initiated by absorbing light energy equal to or more than the band gap of semiconductor photocatalyst [4]. This leads to the excitation of electrons from the valence band (VB) of the semiconductor to their empty conduction band (CB), resulting in the electron–hole pair generation [4]. This photoexcitation process leaves a hole in the VB of the photocatalyst, which can oxidize water of OH^−^ at its surface to produce hydroxyl radical (OH^*^), which is a powerful oxidizing agent and can degrade organic pollutants [12]. Moreover, the pollutants may also be directly oxidized by the holes (h^+^) due to their oxidizing nature, but the detailed reaction mechanism is still under debate. In addition, photoexcited electrons in the CB of a semiconductor can reduce H^+^ ions in aqueous solution to generate hydrogen, or it can produce a superoxide radical anion (O_2_^−^*) by reacting with the dissolved oxygen, hydroperoxide radical (^*^OOH) upon reaction with H^+^ ions [4]. These reactive radical species also have potential to accomplish complete mineralization of the pollutants into H_2_O and CO_2_ [12]. But the main drawback of this process is the instability of the photogenerated species, which can readily recombine with other processes and lose the absorbed energy in the form of heat leading to low photocatalytic efficiency [33]. Therefore, various strategies have been adopted by the scientific community such as heteroatom doping [34], noble metal doping [35], coupling with semiconductors [36] and nanocomposite formation with carbon-based materials, such as graphene [37] and g-C_3_N_4_ [38], to enhance the photocatalytic efficiency. Among the various types of nanocomposites, the materials based on two-dimensional (2D) nanocomposites have attracted particular interest because of their improved properties [39]. It is noteworthy to mention here that various groups have reported zero-dimensional (0D) and one-dimensional (1D) nanocarbon–semiconductor hybrids with excellent photocatalytic efficiency towards pollutant removal and energy generation [29–32]. Hence, the carbon-based nanocomposites with different morphologies have made substantial contribution as promising materials for diverse applications in the field of materials chemistry.

**Figure 1:**
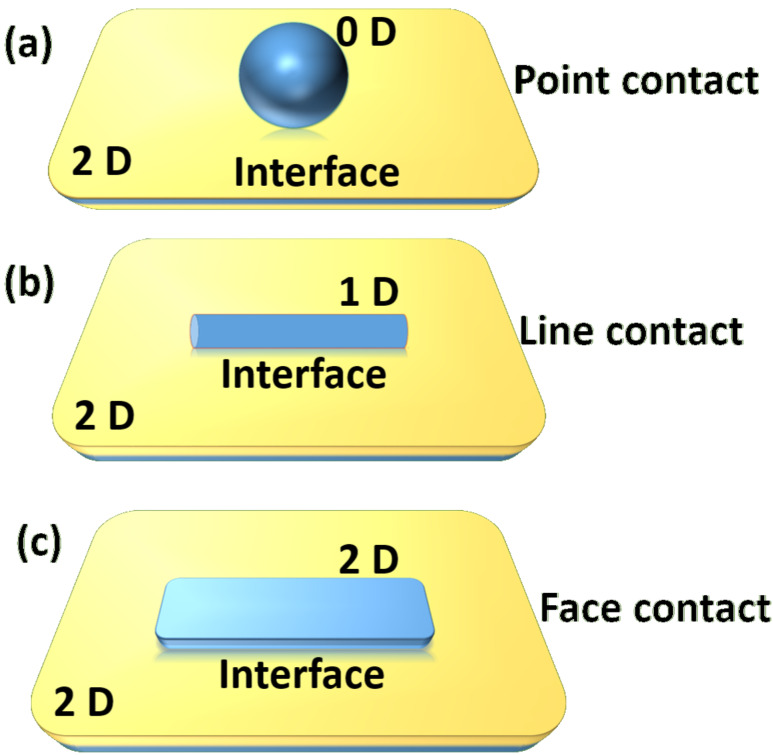
Schematic diagram of interfaces of (a) 0D-2D (b) 1D-2D, and (c) 2D-2D materials.

It has been well-reported in the literature that nanocomposite formation of semiconductors with such 2D materials effectively improves the photocatalytic processes. In addition, these 2D materials possess several extraordinary properties, which makes them more advantageous over other materials as summarized below [39]:

1. high specific surface area with a large number of active sites on the surface to boost photocatalytic reactions as compared to their bulk counterpart;
2. π-conjugated structures, which lead to fast electron transfer and promote the separation of electron−hole pairs on the photocatalyst surface; and
3. excellent support matrix for metals, metal oxide semiconductors and other nanomaterials, which can form efficient heterojunction with intimate contact between them, such as, point-to-face contact (0D-2D), line-to-face contact (1D-2D) and face-to-face contact (2D-2D) as presented in Figure 1. This is more beneficial for the rapid charge transfer and better catalytic dispersion to enhance the photocatalytic activity.

The 2D carbon-based nanomaterials combine several of the above-mentioned advantages of both 2D and carbon-based materials, and have shown great prospects as catalysts for various applications. As this is currently an area of immense research, we decided to write a review article on these materials, especially summarizing the recent developments. Since the scope of 2D carbon-based materials for various applications is very broad as per recent reports on their advances by M. Strano and N. Coleman [40–41], we have focussed our review on only two of the 2D morphology of carbon materials, graphene and g-C_3_N_4_, and their nanocomposites for photocatalytic energy generation and environmental remediation applications. In this review, we firstly discuss the synthetic procedures and salient properties of these two 2D carbon materials, followed by a detailed discussion on what makes them suitable for photocatalysis applications and the different roles played by them during the photocatalysis process. Subsequently, we discuss the use of graphene and g-C_3_N_4_ based nanocomposites for photocatalytic energy generation and environmental remediation applications, along with several recent citations. We then conclude by highlighting the advantages and challenges involved in the use of 2D carbon-based nanocomposites for photocatalysis. Lastly, the future perspectives of research in this field (way ahead) are also briefly discussed.

### Carbon-based 2D materials

#### Graphene

**Figure 2:**
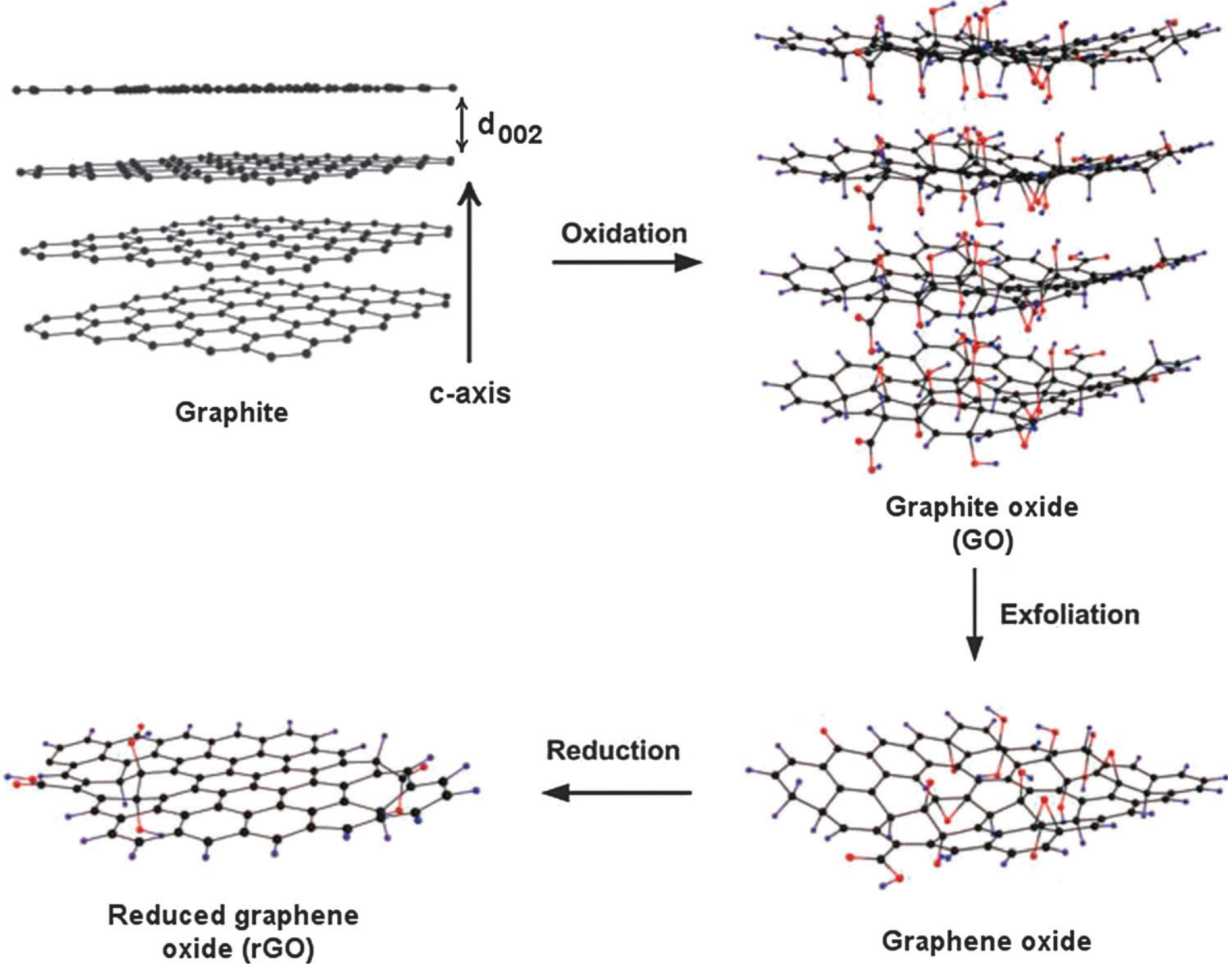
Schematic illustration of the preparation of reduced graphene oxide (RGO) from graphite. Reprinted with permission from [56], copyright 2011 Wiley-VCH.

Since the discovery of graphene in 2004, it has attracted great attention because of its fascinating electrical, thermal, optical and mechanical properties. Basically, graphene consists of a single layer of sp^2^ hybridized carbon atoms densely packed into an atomically thin layer to form a 2D hexagonal honeycomb-like structure [42]. The π-conjugated structure in graphene provides ultrafast electron transfer (200,000 cm^2^·V^−1^·s^−1^), very high specific surface area (2600 m^2^·g^−1^), and high thermal conductivity (5000 W·w^−1^·K^−1^) [43]. In addition to this, graphene possesses high transparency, high elastic modulus (≈1 TPa), high mechanical strength (≈1060 GPa), and optical transmittance (≈97.7%) [44]. These superior properties of graphene make it a potential candidate for technological application such as such as optical electronics [45], photosensors [46] and photocatalysis [47]. As graphene is a zero band gap material and susceptible to oxidative reactions, it is often combined with other semiconductors and metallic nanostructures to form composite materials suitable for various applications, including photocatalysis. Furthermore, due to the exceptional electrical, thermal, optical and mechanical properties, graphene helps to enhance the photocatalytic performance by acting as excellent electron acceptor and transporter in nanocomposites. Moreover, enhanced pollutant adsorption on the surface of graphene is an additional advantage, which accelerates the photocatalytic degradation of adsorbed pollutants [48]. Several chemical and physical methods have been developed for the synthesis of graphene and graphene-based nanocomposites. One of the well-known methods for graphene oxide synthesis is Hummers’ method, which includes chemical oxidation of graphite flakes to form graphene oxide (GO) [49]. GO contains carboxyl, epoxides and hydroxyl groups covalently attached to the graphene sheet. This leads to the loss of electrical conductivity and limits the application of GO in many areas. However, the presence of polar functional groups in GO makes it hydrophilic in nature and it is responsible for the easy dispersal in many solvents such as water, which is helpful for the formation of various composites [50]. The reduction of GO in various reducing conditions forms reduced graphene oxide (RGO) in which electrical conductivity is partly revived. This RGO is also known as chemical-modified graphene [51]. The schematic illustration of RGO preparation from graphite is shown in Figure 2. The composite formation of graphene with semiconductor materials has been reported by various methods, such as hydrothermal/solvothermal [52], sol−gel [53], self-assembly [54], precipitation [55], and photo-reduction [13]. The hydrothermal/solvothermal method for the synthesis of graphene-based nanocomposites involves the treatment of its precursor in a confined volume, teflon-lined autoclave at elevated temperature, wherein high pressure is generated. This method is very important for the synthesis of inorganic nanocrystals and gives rise to highly crystalline nanostructures and also reduces GO to RGO. As the name suggests, water is the main solvent in hydrothermal synthesis method and major advantage of water as the solvent is its abundance in nature as well as its non-toxic, non-carcinogenic and non-flammable nature. However, other solvents like ethanol can also be used as the main solvent in solvothermal method. Hence this method involves a very simple and ecologically-friendly process for the synthesis of nanostructures. By controlling some other parameters, such as concentration, temperature, reaction time, etc., nanocomposites with various exposed crystal facets can be obtained by hydrothermal/solvothermal methods.

The sol–gel method is another widely explored method for the synthesis of graphene-based nanocomposites [53]. The precursor material undergoes a series of reactions, mainly controlled hydrolysis and condensation, to form the desired photocatalyst. The major advantage of using the sol–gel method is the in situ growth of nanostructures so that the various functional groups on the surface of GO sheets are available to provide reactive and anchoring sites for the growth of nanoparticles and hence the resultant photocatalytic materials are chemically bonded with each other [53]. This method has been successfully used in the in situ preparation of various graphene–semiconductor nanocomposites, such as TiO_2_ on GO sheets [57].

Self-assembly is a very important method, wherein micro- and nanostructures assemble spontaneously by supramolecular interactions to form larger functional units [58]. This self-assembly of nanoparticles is very useful for various applications. In the surfactant-assisted ternary self-assembly of metal oxides with functionalized graphene sheets, an anionic surfactant gets adsorbed on the surface of graphene sheets and helps in the dispersion of graphene sheets. Then, the surfactant micelles with graphene sheets bind with metal cations and hence act as building block for self-assembly of metal oxides. Finally metal oxides become crystallized between alternating layers of graphene to form fine layered nanostructures. Self-assembly is also a widely used method for constructing a new class of layered nanostructures with stable, ordered and crystalline structure [58]. In layer-by-layer self-assembly of functionalized graphene nanoplatelets, the electrostatic interactions between graphene nanoplatelets are responsible for self-assembly of graphene sheets. In addition to the above-mentioned methods, there are also other efficient methods for synthesis of graphene–metal oxide hybrid nanocomposites, such as solution mixing [59], UV-assisted reduction [13], microwave irradiation [60] and so on.

#### Graphitic carbon nitride

**Figure 3:**
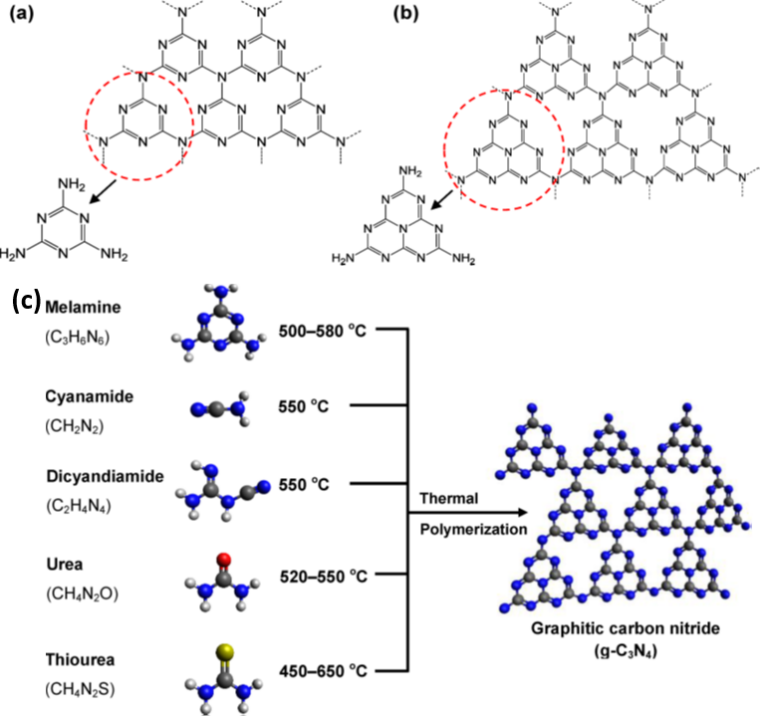
(a) Triazine, (b) tri-s-triazine (heptazine) structures of g-C_3_N_4_, (c) thermal polymerization of different precursors for g-C_3_N_4_ synthesis. Reprinted (adapted) with permission from [68], copyright 2016 American Chemical Society.

The covalent carbon nitride (C_3_N_4_) was discovered by Berzelius with heptazine units as basic structural units [61]. It is reported that C_3_N_4_ possesses seven different phases, viz., α-C_3_N_4_, β-C_3_N_4_, cubic-C_3_N_4_, pseudocubic-C_3_N_4_, g-h-triazine, g-h-heptazine and g-o-triazine, which exhibit the band gaps of 5.49, 4.85, 4.30, 4.13, 2.97, 2.88 and 0.93 eV, respectively [62]. Among these seven phases, the β-C_3_N_4_ crystalline phase possess similar hardness as compared to that of diamond, and the pseudocubic-C_3_N_4_ and g-h-triazine-C_3_N_4_ possess direct band gap structure, while other five phases have indirect band gaps in their bulk structures [62]. It is noteworthy to mention here that the polymeric graphitic carbon nitride (g-C_3_N_4_) has been reported as the most stable, highly ordered polymeric structure with pendant amino groups and tri-s-triazine (C_6_N_7_) as the building structural units (Figure 3a,b) [63]. g-C_3_N_4_ was first reported by Wang et al. in 2009 as an interesting, metal free, n-type semiconductor, polymeric photocatalytic material for the water splitting reaction to evolve H_2_ and O_2_ [64]. The unique optical, electrical and physiochemical properties of g-C_3_N_4_ makes it a multifunctional photocatalytic material [64]. Therefore, g-C_3_N_4_ has attracted immense attention mainly for photocatalytic hydrogen generation reactions and pollutant removal by harvesting visible light due to its suitable band gap energy (≈2.7 eV) [65–66]. Hence this material possesses high photocatalytic efficiency under visible light, which constitutes about 43% of the solar energy spectrum as compared to ultraviolet light (5%). Moreover, the CB and VB of g-C_3_N_4_ are suitably positioned with appropriate potential (CB = −1.13 eV, VB = 1.57 eV), which favours various photocatalytic reactions but mainly hydrogen evolution reactions [67].

The lattice structure of g-C_3_N_4_ is composed of C–N with short interlayer distances and amino functional groups with larger periodic vacancies [67]. In addition to this, g-C_3_N_4_ possesses excellent chemical and thermal stability, unique surface properties with unsaturated N-atoms for anchoring active sites [69]. Furthermore, the stacked 2D layered structure of g-C_3_N_4_ consists of single-layer nitrogen heteroatom-substituted graphite nanosheets, formed through sp^2^ hybridization of C and N atoms, and various layers are bound together by van der Waals forces [69]. Thus it is clear that the lattice structure of g-C_3_N_4_ consists only of two abundant elements, C and N (C/N molar ratio = 0.75), which are earth abundant and nontoxic in nature [61]. More surface active sites, nontoxicity, natural abundance and good thermal stability of g-C_3_N_4_ makes it a multifunctional, sustainable photocatalytic material. The main drawback from which pure g-C_3_N_4_ suffers is poor light absorption and fast recombination of photogenerated electron–hole pairs, which leads to low photocatalytic efficiency and limits its applications [61]. To date, various attempts have been made to improve the light absorption of g-C_3_N_4_ and retard the recombination of photogenerated charge carriers to improve the photocatalytic efficiency. These strategies involve doping with metal atoms [70], non-metal doping [71], coupling with other carbon-based materials [72], and heterojunction formation by coupling with semiconductor materials such as TiO_2_ [73], ZnO [74], CdS [75], SnO_2_ [76], CeO_2_ [77], WO_3_ [78], Fe_2_O_3_ [79], Ag_3_PO_4_ [80], Ag_3_VO_4_ [81], ZnWO_4_ [82], SrTiO_3_ [83], BiVO_4_ [84], Bi_2_WO_6_ [85], BiOX [86–87], etc. These heterojunction formations have proved to be an effective method to improve the separation rate of photogenerated charge carriers to enhance the quantum yield. Notably, such heterojunction formation with semiconductors also enhances the light absorption efficiency of photocatalysts from UV to visible region of the solar energy spectrum.

Furthermore, it is noteworthy to mention here that the surface physicochemical properties of g-C_3_N_4_ can be tuned by introducing impurities into the crystal lattice of polymeric g-C_3_N_4_. Mainly the hydrogen impurities can produce the basic primary and secondary amines on its layer edges [68]. The presence of such basic groups (=NH, −NH_2_) on the surface of g-C_3_N_4_ can remove toxic acidic molecules through electrostatic interactions [68]. The surface hydrophobicity of g-C_3_N_4_ can be changed by chemical oxidation by introducing various hydroxyl and carbonyl groups, which eventually lead to good dispersion during catalytic process. The layered g-C_3_N_4_ exhibit excellent chemical stability and is insoluble in various kinds of acid, base and organic solvents like toluene and THF [68]. The good chemical and thermal stability of carbon nitride permits its use in PEC cells even under oxygen atmosphere [63]. Furthermore, the chemical inertness and insolubility of g-C_3_N_4_ in most of the known solvents is one main hurdle for easy synthesis of its g-C_3_N_4_ based nanocomposites. Recently, layered g-C_3_N_4_ based nanocomposites have attracted much attention because of reports on some simple synthesis methods [68]. The g-C_3_N_4_ and its nanocomposites with semiconductors and carbon-based materials can be easily designed and synthesized by thermal condensation of several low cost, solid precursor materials such as urea, thiourea, dicyandiamide, cyanamide and guanidine hydrochloride at high temperature (500–600 °C) in air or inert atmosphere (Figure 3c) [88–90]. It is noteworthy to mention here that by using different precursor materials, some of the properties, such as microstructure, adsorption affinity and isoelectric point of g-C_3_N_4_ can be tuned [91]. It is known that catalysis is a surface phenomenon, which is affected by the surface structure and morphology of catalytic material. Therefore the fabrication of g-C_3_N_4_ with different microstructures is expected to show different surface properties and ability to enhance the photocatalytic performance. As per one of the reports by Zhu et al., g-C_3_N_4_ synthesized by using melamine, thiourea, or urea as precursor, exhibited different microstructure and isoelectric points [91]. The g-C_3_N_4_ prepared by the thermal condensation method generally exhibit low surface area, which can limit its practical applications, as high specific surface area of catalyst is highly desirable for enhanced photocatalytic activity [92]. Therefore, the preparation of exfoliated thin g-C_3_N_4_ nanosheets is becoming one of interesting areas for further exploration of the potential of g-C_3_N_4_ in various photocatalytic applications [65]. In addition to the thermal condensation method, there are also some other strategies reported for the preparation of g-C_3_N_4_ based nanocomposites, which includes molecular self-assembly [93], microwave assisted heating [38], molten salt synthesis [94] and ionic liquid strategy [95].

### 2D carbon-based nanocomposites as photocatalysts

#### 2D graphene-based photocatalysts for energy generation

**Figure 4:**
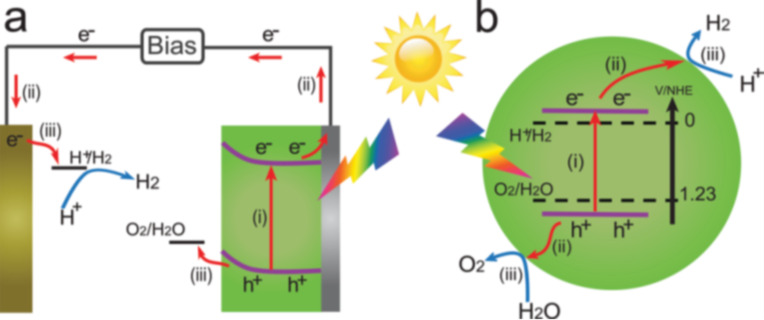
The principle of (a) photoelectrochemical water splitting and (b) photocatalytic water splitting for H_2_ generation. Reprinted with permission from [102], copyright 2013 Wiley-VCH.

Photocatalytic H_2_ production through solar water splitting has been widely explored as it has several advantages like easy and abundant availability of raw materials, tunable electronic structure and the fact that combustion of hydrogen in air produces water; hence, this method is ecologically-friendly [96]. Moreover the H_2_ production has attracted great attention as a renewable, sustainable energy source due to growing environmental issues [96–97]. Therefore photocatalytic water splitting has been extensively studied using various semiconductor-based materials and many new semiconductor-based photocatalysts have been successfully developed and investigated recently [4,98–99]. In 1972, Fujishima and Honda achieved photoelectrocatalytic water splitting using a TiO_2_ electrode [6]. TiO_2_ was irradiated with UV light and electrons and holes are generated in the CB and VB, respectively. The TiO_2_ electrode acts as an anode and is connected to a Pt cathode. The photogenerated electrons reduce H^+^ ions to generate H_2_ on the Pt electrode while holes oxidize water to form O_2_ on TiO_2_ electrode, as illustrated in the Figure 4a. After this discovery, semiconductor-based materials with suitable band gaps have attracted much attention in this field. In order to efficiently utilize the solar energy, many photoelectrochemical cells have been developed for hydrogen production [100–101]. Basically, in the process of photocatalytic water splitting, photons with energy greater than the band gap energy of the chosen semiconductor material result in the formation of photogenerated electrons and holes in the conduction band (CB) and the valence band (VB), respectively. These photogenerated electron–hole pairs are responsible for the reduction and oxidation reactions, i.e., reduction of H^+^ → H_2_ in CB and oxidation of H_2_O → O_2_ in the VB, as illustrated in Figure 4b [4,102].

**Figure 5:**
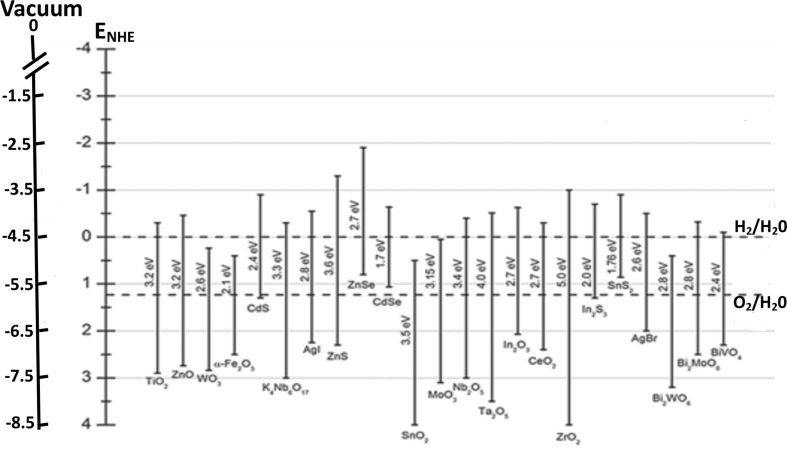
Band gaps and band positions of a) n-type semiconductors and b) p-type semiconductors used for nanocomposite photocatalyst heterojunctions. Reprinted (adapted) with permission from [103], copyright 2013 Wiley-VCH.

The most important point in achieving water splitting is the position of the VB and CB in semiconductor materials. The bottom level of the CB must be more negative than the redox potential of H^+^ → H_2_ (0 V vs NHE, where NHE refers to the normal hydrogen electrode), while the top level of the VB must be more positive than the oxidation potential of H_2_O → O_2_ (1.23 V vs NHE) [4]. Therefore 1.23 eV is the minimum band gap for water splitting and this band gap corresponds to light at 1008 nm (near-infrared region). According to standard literature [4], the wavelength and eV are related to each other as, band gap (eV) = 1240 / λ (nm). Hence a suitable band gap value plays a crucial role in order to make the catalytic material active in the visible region of light to generate H_2_ and O_2_ by water splitting. The band gap of some semiconductor materials with band positions are summarized in Figure 5 [103].

As it is well known, the band gap and wavelength are directly related to each other, and suitable band gap engineering is required to make photocatalysts active in the visible light region of the spectrum. The overall water splitting reaction on the surface of a semiconductor material occurs in three main steps, (1) absorption of light, (2) charge separation, (3) redox reactions on the catalyst surface.

The first step involves the absorption of light by the photocatalyst and generation of electron–hole pairs in the CB and VB. The second step involves the charge separation and migration of charge carriers to the surface. Higher crystallinity and smaller size of particles play a significant role in enhancing the photocatalytic activity by decreasing the recombination probability of photogenerated charge carriers [4]. It is well known that higher crystallinity leads to enhanced photocatalytic activity. Finally, the third step involves the reduction and oxidation of adsorbed species at the different reaction sites, wherein hydrogen production takes place by the reduction of H^+^ ions in the CB. Hydrogen evolution by water splitting is promoted by the presence of cocatalysts, such as Pt, Rh, NiO, and RuO_2_. These cocatalysts are mainly helpful to introduce the active sites on the photocatalyst surface, to facilitate the electron transfer from the CB of excited semiconductor, and hence, to enhance the process of H_2_ generation [11]. However, the sacrificial agents (methanol, ethanol, sodium sulphide, sodium sulphite, etc.) are always employed in photocatalytic water splitting reactions to scavenge holes and hence suppress photogenerated charge recombination effectively. When graphene-based nanocomposites are used as photocatalysts for energy generation through the water splitting reaction, the graphene in the nanocomposite plays different roles, such as photocatalyst, cocatalyst, electron acceptor/transporter and photosensitizer. These roles are described in detail in the following sections.

#### Graphene as a photocatalyst

A photocatalyst is a substance which produces the catalytic activity using energy from light without undergoing any change in itself [104]. The photocatalytic activity depends on the generation of electron–hole pairs in the catalyst under the influence of light energy [105]. These photogenerated charge carriers then generate free radicals such as hydroxyl, superoxide, hydroperoxide, which migrate to the surface of the catalyst and undergo secondary reactions [106]. Due to the superior properties of 2D layered materials, particularly high specific surface area, ultrafast electron transfer and better dispersion, such materials have been investigated in detail by various research groups. Hence, a new class of photocatalysts with significantly suppressed charge recombination and fast interfacial charge transfer have been developed using these materials with extraordinary H_2_ evolution capability.

**Figure 6:**
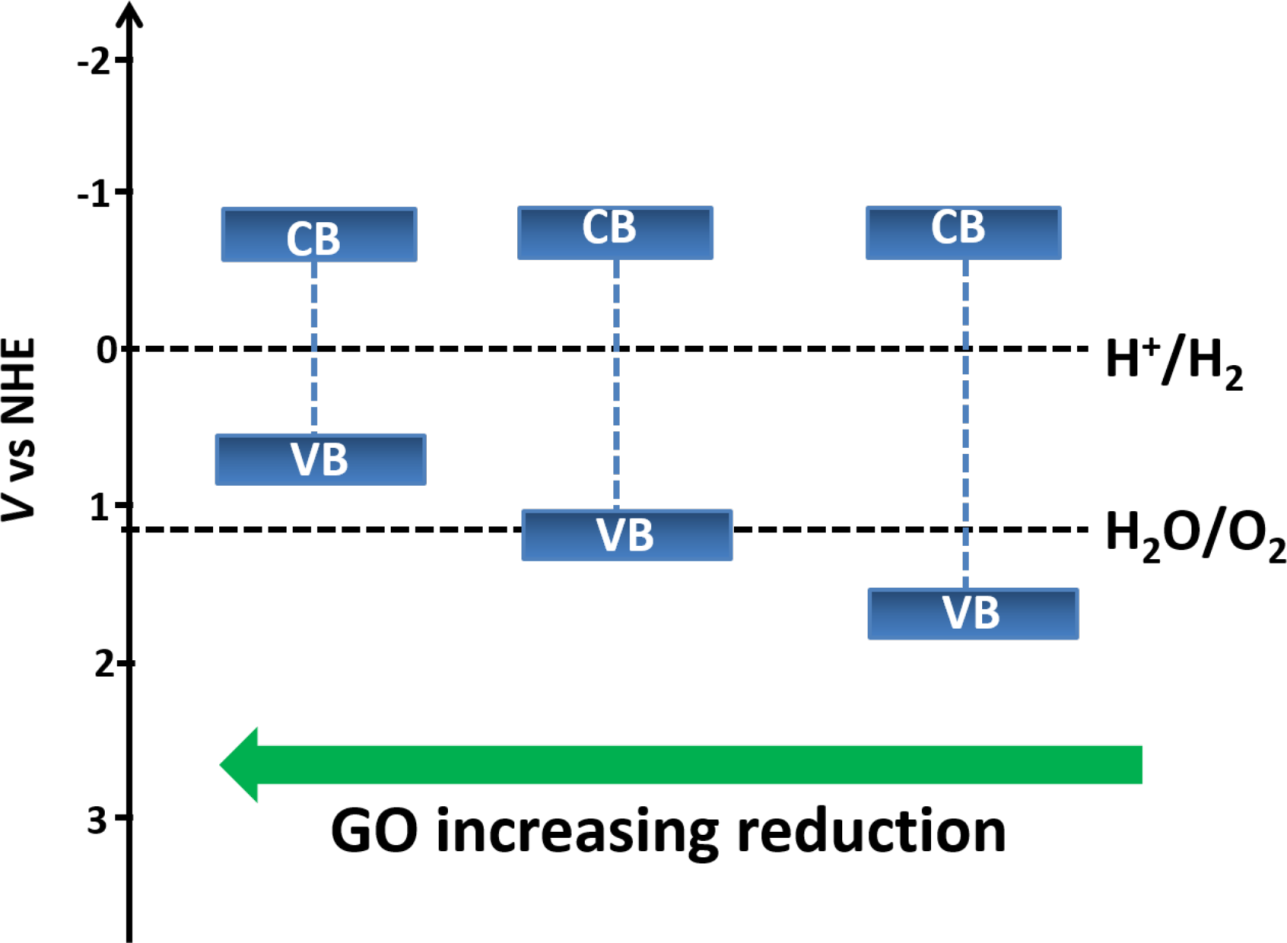
Energy level diagrams of GO with different degrees of reduction in comparison with the potentials for water reduction and oxidation.

Yeh et al. [107] demonstrated graphite oxide as a photocatalyst for hydrogen generation from water without using any noble metal as a cocatalyst. They used moderately oxidized GO with a band gap in the range 2.4–4.3 eV, which can absorb visible light. The oxidation of graphite introduces many oxygen-containing functional groups such as carboxyl, epoxide and hydroxyl groups on its surface, which make GO hydrophilic. Thus GO is easily dispersible in water and hence it has more exposed area in aqueous solutions and effectively catalyses the water splitting reaction. In addition, the band gap of GO can be tuned with its degree of reduction. The variation of the band gap of GO with increasing degree of reduction has been illustrated in Figure 6. Its electrical conductivity decreases with increasing oxidation level, meaning fully oxidized GO acts as an insulator and partially oxidized GO acts as a semiconductor [108]. The conduction band edge of GO is mainly formed by the anti-bonding π* orbital which has a higher energy level of −0.52 eV. Thus, due to the more negative anti-bonding π* orbital, which is needed for hydrogen generation, GO can act as a photocatalyst. Also, the VB edge of GO is mainly composed of O 2p orbitals and may not be positive enough to oxidize water but it varies with the reduction degree. It has been observed that the band gap of GO decreases with the reduction. It is well-reported in the literature that for GO with 12.5% of the oxygen atoms, the top energy level of the VB is not high enough to oxidize water for O_2_ evolution; but at the same time, for GO having 25% coverage of oxygen atoms, the energy level of the CB is high enough for O_2_ evolution from water [109–110]. Hence, by tuning the electronic properties of GO, it can act as a promising material for H_2_ generation from water without any cocatalyst. The possible mechanism of water splitting with GO as a photocatalyst, using methanol as hole scavenger, can be summarized as [107],


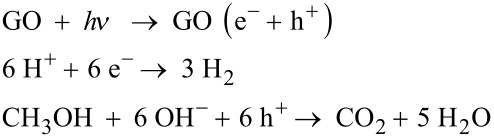


Eda et al. have investigated the insulator → semiconductor → semimetal transition in RGO with degree of reduction [111]. They found that the energy gap even approaches zero with the extensive degree of reduction. Therefore, this possibility of band gap engineering of RGO is always an area of interest for its implementation in various applications. Yeh et al. [112] also demonstrated the photocatalytic activity of GO in hydrogen and oxygen evolution from water with different oxidation levels. They showed that the band gap energy of GO increases with the increasing oxidation level of GO, which limits the light absorption. This, instead of the fact that GO has a narrow band gap energy, is the main contributor to the poor photocatalytic activity. It was also observed that during the photocatalytic reaction, the H_2_ evolution rate was constant. This is mainly because the GO band gap decreases during the reaction, leading to the upward shift of the VB. Teng et al. [113] have shown the functional engineering of GO for tuning its band gap by its treatment with ammonia and have explored its photocatalytic activity in water splitting reactions under visible light irradiation. Ammonia-modified GO (NGO) shows n-type conductivity due to the introduction of nitrogen functionality. The band gap of NGO is narrowed due to the removal of various epoxy and carboxyl groups and it further acts as a promising photocatalyst towards the H_2_ and O_2_ generation from water splitting.

#### Graphene as a cocatalyst

A cocatalyst is a substance which assists the catalyst in a chemical reaction and hence enhances the activity of the catalyst [114]. Cocatalysts are generally loaded on the surface of semiconductors as a dispersion of nanoparticles and accelerate the photocatalytic rate by introducing more reaction sites and promoting charge separation in semiconductors [115]. In water splitting reactions, generally noble metals (e.g., Pt, Rh) and some metal oxides (e.g., NiO) act as the cocatalyst and these are loaded on the surface of photocatalysts to produce more reactive sites and to reduce the activation energy for H_2_ and O_2_ gas evolution. Cocatalysts also enhances the charge separation in photocatalytic materials because of their high work function. This high work function of noble metals and some metal oxides accelerates the transfer of electrons from the CB of excited semiconductors to the cocatalyst and results in the formation of a Schottky barrier, which efficiently decreases the recombination of charge carriers [102]. Hence cocatalysts play a crucial role in the enhancement of photocatalytic activity by providing abundant reaction sites for H_2_ evolution, increasing interfacial charge transfer and reducing the recombination probability of photogenerated electron–hole pairs [116]. However, the high cost of noble metals limits their use as cocatalysts on a large scale. Graphene has been demonstrated to be one of the best alternatives for noble metals. Graphene acts as a promising cocatalyst in H_2_ evolution reactions due to its high work function (4.42 eV) [117], and the reduction potential of graphene/graphene^−^ is reported to be −0.08 eV, which is more negative than reduction potential of H^+^ → H_2_ [52]. It is noteworthy to mention here that the work function of any material is an important parameter for many technical applications, mainly device fabrication as it decides contact properties with foreign material and charge transfer direction in nanocomposites. The work function of carbon-based materials, graphene, GO, carbon nanotubes (CNT) and g-C_3_N_4_ has been presented in Table 1.

**Table 1:** Work function of carbon-based materials.

Sl. no.	Material	Work function (eV)	Ref.

1	graphene oxide	3.7–5.1	[118]
2	reduced graphene oxide	4.5	[119]
3	graphene	4.8–5.1	[120]
4	graphitic carbon nitride	4.4–4.7	[121]
5	carbon nanotubes	4.7–4.9	[122]

**Figure 7:**
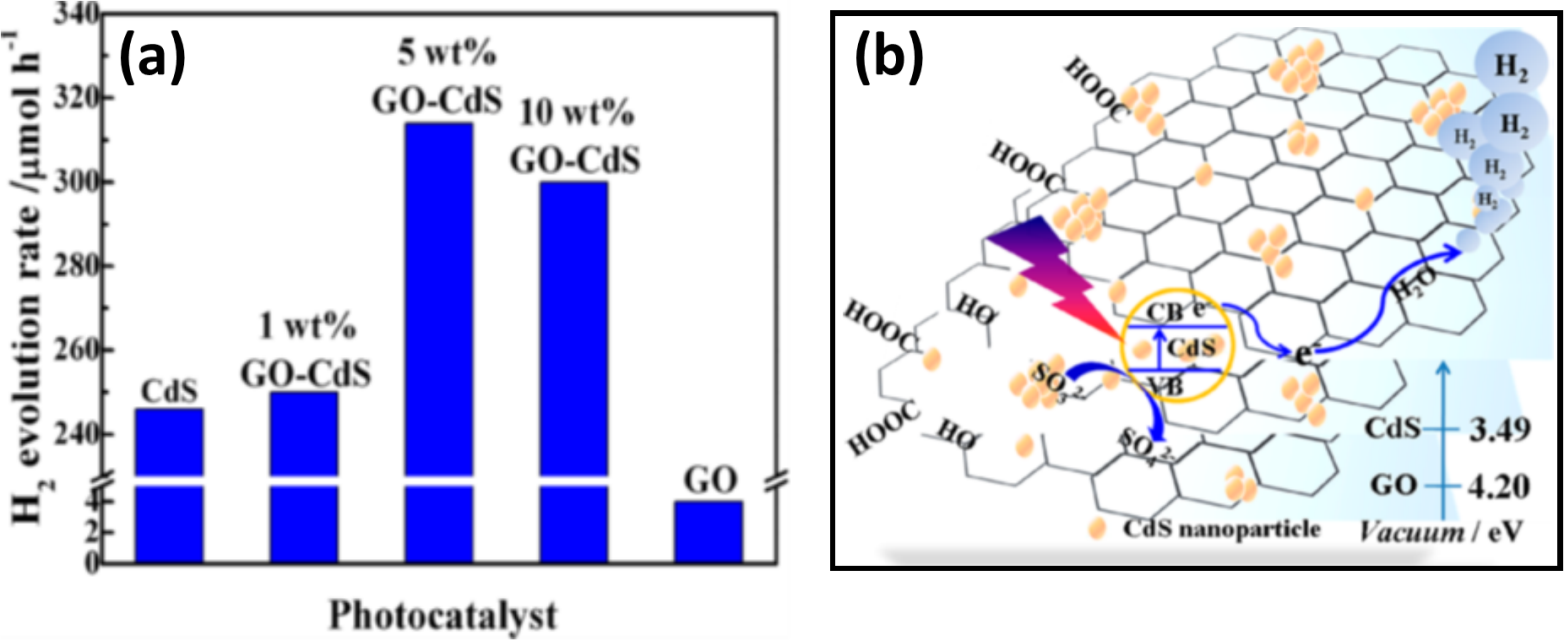
(a) Comparative H_2_ production rate over various GO–CdS nanocomposites under visible light irradiation. (b) The mechanism of H_2_ production over the GO–CdS nanocomposite. Reprinted (adapted) with permission from [123], copyright 2012 American Chemical Society.

The role of graphene as a cocatalyst has been investigated by various research groups. Peng et al. [123] reported graphene oxide (GO)–CdS nanocomposites for photocatalytic hydrogen evolution by using Na_2_S and Na_2_SO_3_ as sacrificial agents, where GO acts as a supporting matrix for the CdS nanoparticles, which are about 10 nm in size. Due to the narrow band gap CdS is active in the visible region. They observed the highest H_2_ production rate of 314 µmol h^−1^ for the composition having 5 wt % of GO, as can be seen in Figure 7a. Herein, GO functions as an excellent electron acceptor and transporter from the CB of excited CdS to reaction sites. Thus graphene reduces the recombination rate of photogenerated charge carriers and improves the interfacial charge transfer process, which is ultimately responsible for the enhanced activity of the photocatalyst. The general mechanism for this reaction has been illustrated in Figure 7b. A similar binary nanocomposite has been reported by Xiang et al., which consists of graphene-modified TiO_2_ nanosheets [124]. This composite shows excellent H_2_ production rate of 736 µmol h^−1^ with 1 wt % of graphene content. Here graphene also plays a key role as the cocatalyst to enhance the H_2_ production.

**Figure 8:**
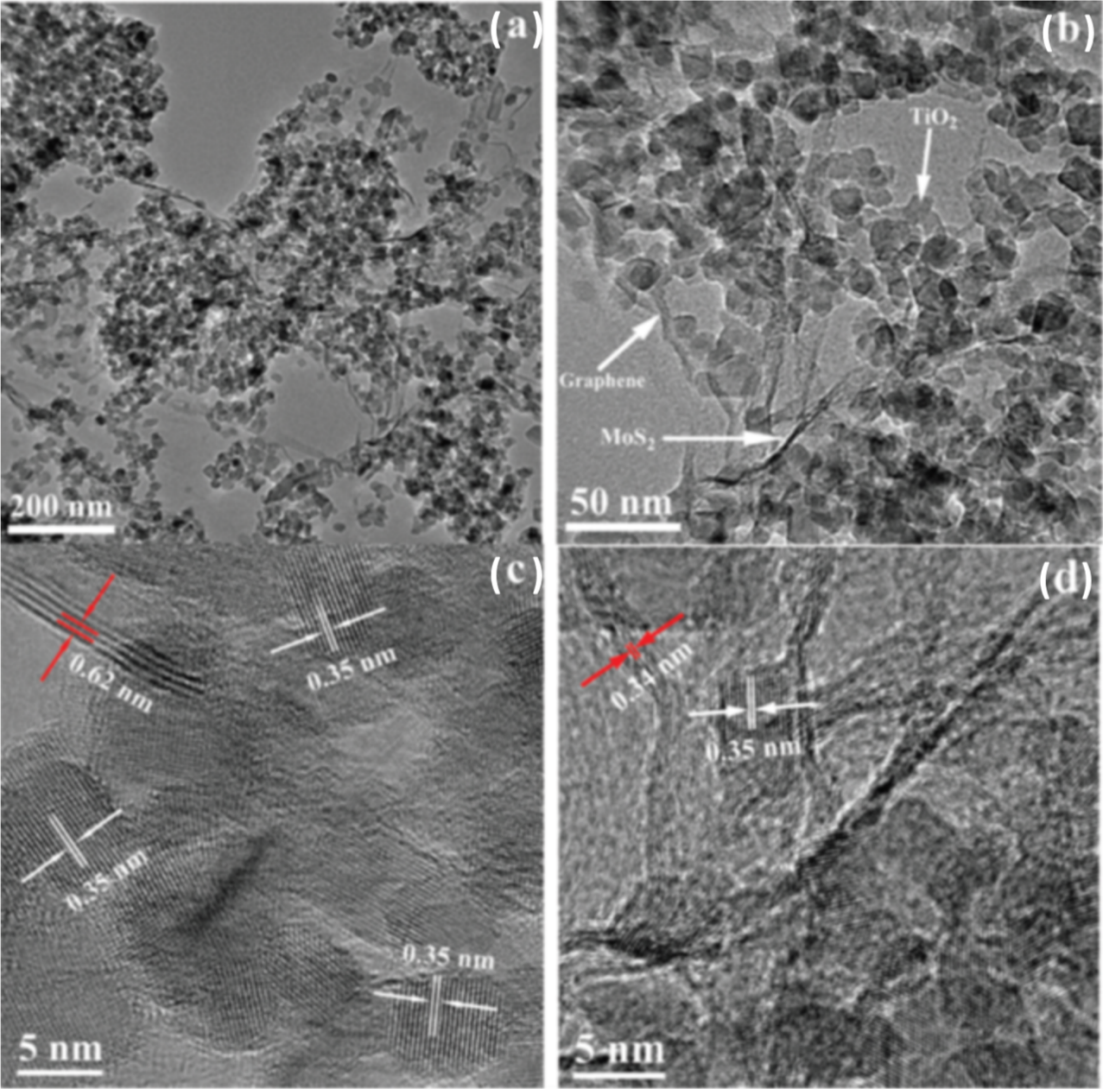
(a,b) TEM images of TiO_2_–MoS_2_–graphene composites and (c,d) high-resolution TEM images of TiO_2_–MoS_2_–graphene composites. Reprinted with permission from [126], copyright 2012 American Chemical Society.

**Figure 9:**
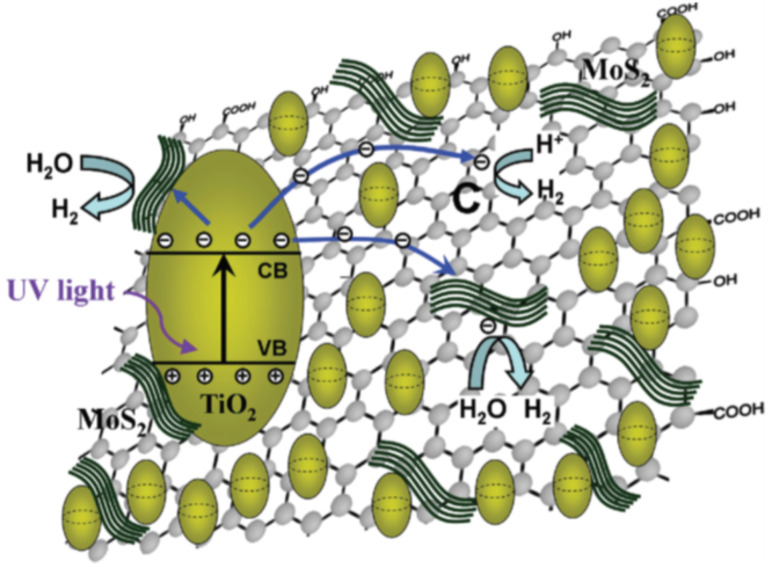
Proposed mechanism for the photocatalytic H_2_ generation over TiO_2_–MoS_2_–graphene composite. Reprinted with permission from [126], copyright 2012 American Chemical Society.

Furthermore, Lv et al. [125] demonstrated the cocatalytic function of metal-doped graphene (Cu-doped graphene–TiO_2_ composites). They found the H_2_ generation efficiency of Cu-graphene cocatalyst is about five times higher than pure graphene cocatalyst. Similarly some other groups have also investigated the cocatalytic role of graphene, for example Ye et al. [116] have reported CdS–MoS_2_–graphene nanocomposites, which is active in visible light for hydrogen generation. They reported the hydrogen evolution rate of 1.8 mmol h^−1^ in lactic acid solution at 420 nm, which is much higher than that of the Pt–CdS system in the same solution. This high H_2_ evolution rate was mainly achieved because of the excellent cocatalytic function of MoS_2_–graphene, which leads to the higher number of reaction sites and fast charge transfer. Moreover, in nanometer-sized MoS_2_, exposed S atoms have strong affinity to H^+^ ions in solution, which are reduced to H_2_ by transferred electrons from the CB of CdS. Similarly, a noble-metal-free, ternary nanocomposite of TiO_2_–MoS_2_–graphene has been reported by Yu et al. for H_2_ generation [126]. This composite prepared by a two-step hydrothermal process lead to uniform dispersion of TiO_2_ nanopartilces over layered MoS_2_–graphene (MG), as shown in Figure 8. Herein, the MG hybrid plays a crucial role for charge separation in UV-excited TiO_2_ nanoparticles and the observed hydrogen production rate was 165 µmol h^−1^ for the composition having 0.5 wt % of MG hybrid. Figure 9 presents the proposed mechanism for the enhanced electron transfer in the TiO_2_–MG system under UV irradiation showing the photoexcited electron transfer from the CB of TiO_2_ to the MoS_2_ nanosheets, followed by transfer to graphene sheets, wherein H_2_ is produced from H^+^ ions.

#### Graphene as a photosensitizer

Apart from the photocatalytic and cocatalytic role of graphene, it is worth to discuss the photosensitizer role played by graphene in many nanocomposite materials. A photosensitizer is a light-absorbing substance which mediates reactions either in living cells or in chemical systems [127]. So far graphene–semiconductor-based composites have been widely explored for H_2_ generation in which mainly graphene acts as the electron acceptor and transporter, and hence, enhances the life span of photogenerated charge carriers, which leads to improved H_2_ evolution. Besides this, graphene can act as an excellent photosensitizer for semiconductors in nanocomposites [128]. The role of graphene as a photosensitizer has been proved theoretically as well as experimentally [128–129].

The photosensitizer role of graphene has been demonstrated by Du et al. [129] on graphene–titania hybrid nanocomposites and explained as the interfacial charge transfer by using density functional calculations. They demonstrated the formation of a charge transfer complex at the interface of graphene and titania due to the work function difference of both materials and upon visible light irradiation, the electrons in the upper VB of graphene can be excited to the CB of titania. As TiO_2_ is inactive under visible light irradiation, the photoactivity was mainly attributed to the photosensitizer, graphene, which absorbs light to generate the charge carriers, which are then utilized to reduce the adsorbed species on the surface of photocatalyst. Zhang et al. [130] also explored the photosensitizer role of graphene by reporting the nanometer-sized assembly of ZnS on graphene sheets and the interfacial contact between them. They formulated a new photocatalytic mechanism for this visible-light-based activity of this nanocomposite. As ZnS is not active under visible light, the light must be absorbed by graphene to produce photogenerated electrons, which gets transferred to the CB of ZnS, thereby making the wide band gap semiconductor visible light active. Peng et al. fabricated TiO_2_–graphene binary nanocomposites by a simple hydrothermal method and demonstrated the high visible-light-based H_2_ evolution from water [131]. Herein, they claimed graphene as the photosensitizer and efficient interfacial charge transfer was observed upon visible light irradiation. Hence, on the basis of all the above-mentioned reports, it can be concluded that besides acting as an electron reservoir to capture and shuttle the electrons, graphene also act as a photosensitizer and transform the UV-active semiconductors into visible light responsive materials. This photosensitization by graphene has opened many new paths in fabricating novel graphene–semiconductor-based nanocomposites for various photocatalytic applications. In addition to the reports cited above, several graphene-based nanocomposites have been successfully developed and utilized for photocatalytic energy generation applications. Some of the noteworthy recent ones have been summarized in Table 2.

**Table 2:** Photocatalytic energy generation using graphene-based nanocomposites. GR – graphene; RGO – reduced graphene oxide; LED – light emitting diode; SS – solar simulator; TEOA – triethanolamine.

Sl no.	Photocatalyst	Synthesis route	Light source	Sacrificial reagents	H_2_ production	Ref. (year)

1	RGO–TiO_2_	sol–gel	500 W Xe lamp	Na_2_S and Na_2_SO_3_	8.6 µmol h^−1^	[57] (2013)
2	GR–TiO_2_	sol–gel	500 W Xe lamp	Na_2_S and Na_2_SO_3_	17.2 µmol	[53] (2010)
3	RGO–TiO_2_	hydrothermal	UV	Na_2_S and Na_2_SO_3_	20 µmol h^−1^	[132] (2011)
4	RGO–TiO_2_ (P25)	hydrothermal	200 W Xe arc lamp	–	74 µmol h^−1^	[13] (2011)
5	GR–CdS	solvothermal	350 W Xe lamp	lactic acid	1.12 mmol h^−1^	[133] (2011)
6	RGO–Cu_2_O	in situ growth	150 W Xe lamp	methanol	264.5 µmol h^−1^ g^−1^	[134] (2012)
7	GR–Cu–TiO_2_	hydrothermal and photodeposition	300 W Hg lamp	–	10.2 mmol	[125] (2012)
8	GO–CdS	precipitation process	300 W Xe lamp	Na_2_S and Na_2_SO_3_	314 µmol h^−1^	[123] (2012)
9	RGO–Zn*_x_*Cd_1−_*_x_*S	coprecipitation - hydrothermal reduction	SS (AM 1.5 G)	Na_2_S and Na_2_SO_3_	1824 µmol h^−1^ g^−1^	[52] (2012)
10	RGO–MoS_2_	hydrothermal	300 W Xe lamp	TEOA	83.8 µmol h^−1^	[135] (2012)
11	RGO–CdS–ZnO	solid state	500 W tungsten halogen lamp	Na_2_S and Na_2_SO_3_	751 µmol h^−1^ 0.2 g^−1^	[136] (2012)
12	GR–TiO_2_–MoS_2_	hydrothermal	UV	ethanol	165.3 µmol h^−1^	[126] (2012)
13	RGO–N–TiO_2_	hydrothermal	UV–visible	methanol	716 µmol h^−1^ g^−1^ 112 µmol h^−1^ g^−1^	[137] (2013)
14	GR–MoS_2_–CdS	hydrothermal	300 W Xe lamp	lactic acid	2.32 mmol h^−1^	[138] (2014)
15	GR–MoS_2_–CdS	hydrothermal	300 W Xe lamp	Na_2_S and Na_2_SO_3_	1.8 mmol h^−1^	[116] (2014)
16	GR–Au–TiO_2_	microwave-assisted hydrothermal	LED lamp (420 nm)	–	296 µmol h^−1^ g^−1^	[139] (2014)
17	GR–MoS_2_–ZnS	hydrothermal	300 W Xe lamp	Na_2_S and Na_2_SO_3_	2258 µmol h^−1^ g^−1^	[140] (2014)
18	GR–Au–TiO_2_	hydrothermal and Photodeposition	450 W Hg lamp	methanol	1.34 mmol	[141] (2014)
19	GO-reduced TiO_2_	laser ablation in liquid	SS (AM 1.5G)	–	16 mmol h^−1^ g^−1^	[142] (2016)
20	GR–CdS	solvothermal	300 W Xe lamp	–	175 µmol h^−1^	[143] (2016)
21	RGO–Pt–TiO_2_	step-wise	SS (AM 1.5G)	TEOA	1075.68 µmol h^−1^ g^−1^	[144] (2017)

#### 2D g-C_3_N_4_-based photocatalysts for energy generation

The development of g-C_3_N_4_-based photocatalysts for water splitting reactions requires several important factors to be taken into account. First of all, the enhanced light absorption capability and effective heterojunction is used to separate electron–hole pairs during photocatalytic process. Next, the CB and VB potentials of the semiconductor should be appropriately positioned to favour H_2_ evolution and O_2_ evolution by water splitting reaction, by charge transfer as per favoured potential. Since it is not possible for a bare g-C_3_N_4_ to fulfil all these requirements, nanocomposite formation with metal oxide semiconductors, metals and other carbon-based materials is always a preferable route for designing photocatalytic materials with desired properties. The nanocomposite heterojunctions can drastically enhance the photocatalytic efficiency by enhanced light absorption in combination with narrow band gap semiconductors, cocatalytic effect, which results in and the formation of a p–n heterojunction or Schottky junction, which can effectively suppress the photogenerated charge carrier recombination and facilitate their transfer.

As mentioned earlier, g-C_3_N_4_ was first investigated as a photocatalyst by Wang et al. [64] in 2009 for visible-light-based water splitting reactions to generate clean, renewable energy in the form of H_2_. They found and explained the appropriate band gap structure of g-C_3_N_4_ to absorb visible light and evolve H_2_ and O_2_ by reduction and oxidation reactions during the photocatalytic process. After this report, several research groups performed dedicated studies on g-C_3_N_4_ and its nanocomposites to generate H_2_ by photocatalytic process. Recently, the coupling of g-C_3_N_4_ with various metal oxides/sulfides, composite oxides, BiOX halides (X = Cl, Br, I), AgX, noble metals and graphene has attracted great attention for the formation of heterojunctions with excellent light absorption and charge transfer kinetics, which is discussed in the following sections of this article.

#### g-C_3_N_4_-oxide/sulfide nanocomposites

**Figure 10:**
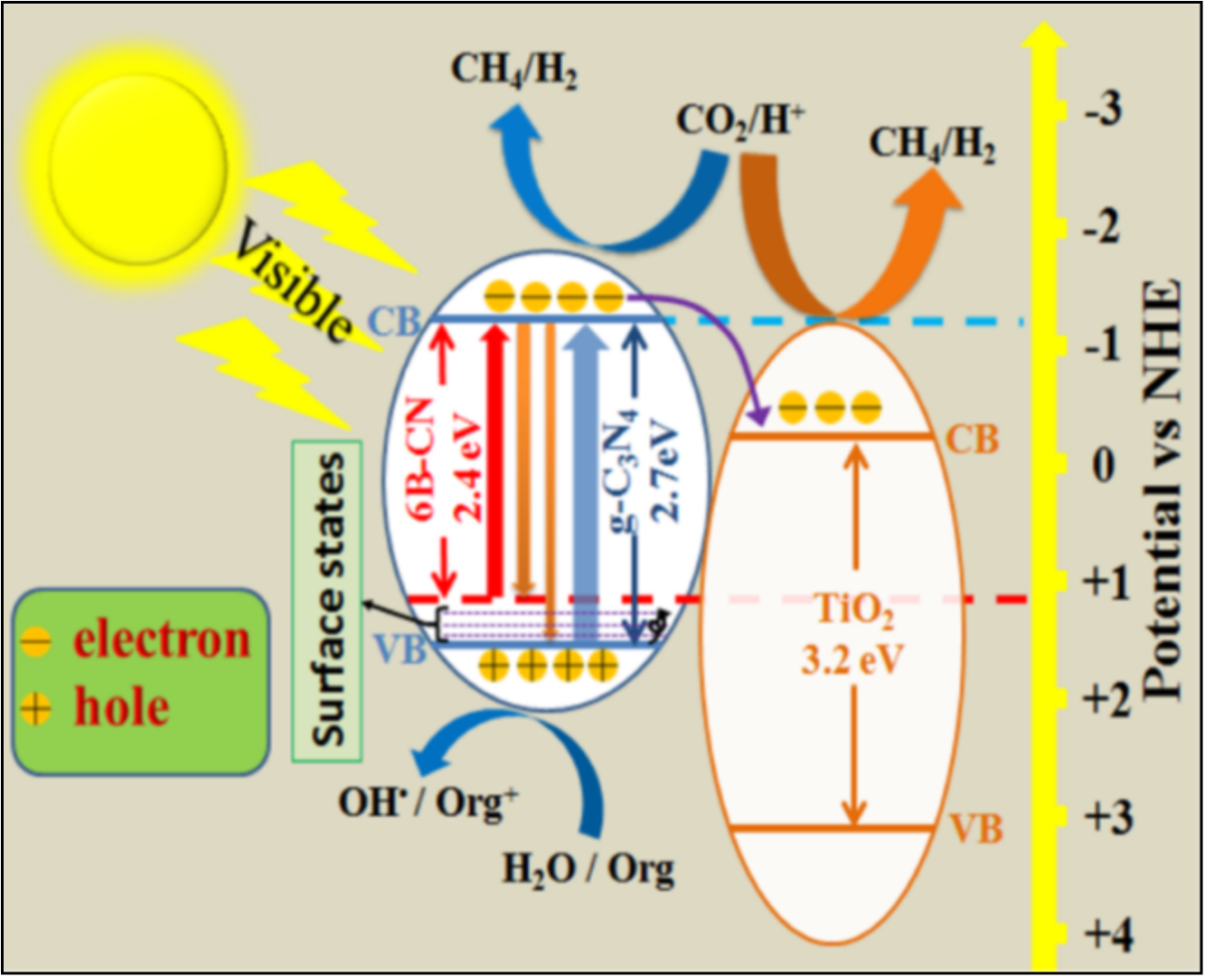
Proposed mechanism of BCN-T system under visible irradiation for H_2_ generation, pollutant removal and carbon dioxide reduction. Reprinted with permission from [145], copyright 2015 American Chemical Society.

Jing et al. [145] reported the cocatalyst-free boron-doped g-C_3_N_4_–TiO_2_ (BCN-T) nanocomposite for H_2_ generation from CH_3_OH under visible light irradiation. The boron doping in g-C_3_N_4_ nanosheets introduces the impurity near to the VB top level, which traps holes and hence the photoinduced electrons were transferred from the CB of g-C_3_N_4_ to the CB of TiO_2_ as per their band potentials (Figure 10), which further leads to the photocatalytic reaction for fuel production. Hence the synergetic effect of boron doping and heterojunction formation with TiO_2_ results in the greatly enhanced, photogenerated charge transfer results with a 29-fold higher H_2_ production as compared to the bare g-C_3_N_4_. Thus this study demonstrates the fabrication of low cost, highly efficient g-C_3_N_4_ nanosheet-based nanocomposites with improved light absorption and charge transfer to generate clean energy.

Shi et al. reported the visible-light responsive g-C_3_N_4_-InVO_4_ nanocomposite heterojunction by in situ growth of InVO_4_ nanoparticles onto the surface of g-C_3_N_4_ nanosheets by a hydrothermal synthesis process [146]. The g-C_3_N_4_ nanosheet serves as an excellent support matrix for the in situ growth of nanoparticles, which were 20 nm in diameter and the interface formation between the two semiconductors improves charge transfer across the interface by inhibiting recombination. The H_2_ evolution rate of 212 µmol h^−1^ g^−1^ was achieved with this nanocomposite material.

Feng et al. reported novel CdS quantum dot (QDs) coupled with g-C_3_N_4_ photocatalysts by a chemical impregnation method [16]. The reported photocatalyst was used for visible-light-based H_2_ evolution from an aqueous methanol solution with Pt as a cocatalyst. The effect of CdS loading was optimized to be 30 wt % of the photocatalyst. The optimized catalyst achieved about a nine times higher H_2_ evolution rate of 17.27 μmol h^−1^, as compared to pure g-C_3_N_4_. The improved photocatalytic H_2_ evolution by the CdS–g-C_3_N_4_ nanocomposite has been attributed to the synergistic effect of g-C_3_N_4_ and CdS QDs, which leads to the efficient separation of the photogenerated charge carriers and thereby enhances the visible light photocatalytic H_2_ production activity of the nanocomposite.

**Figure 11:**
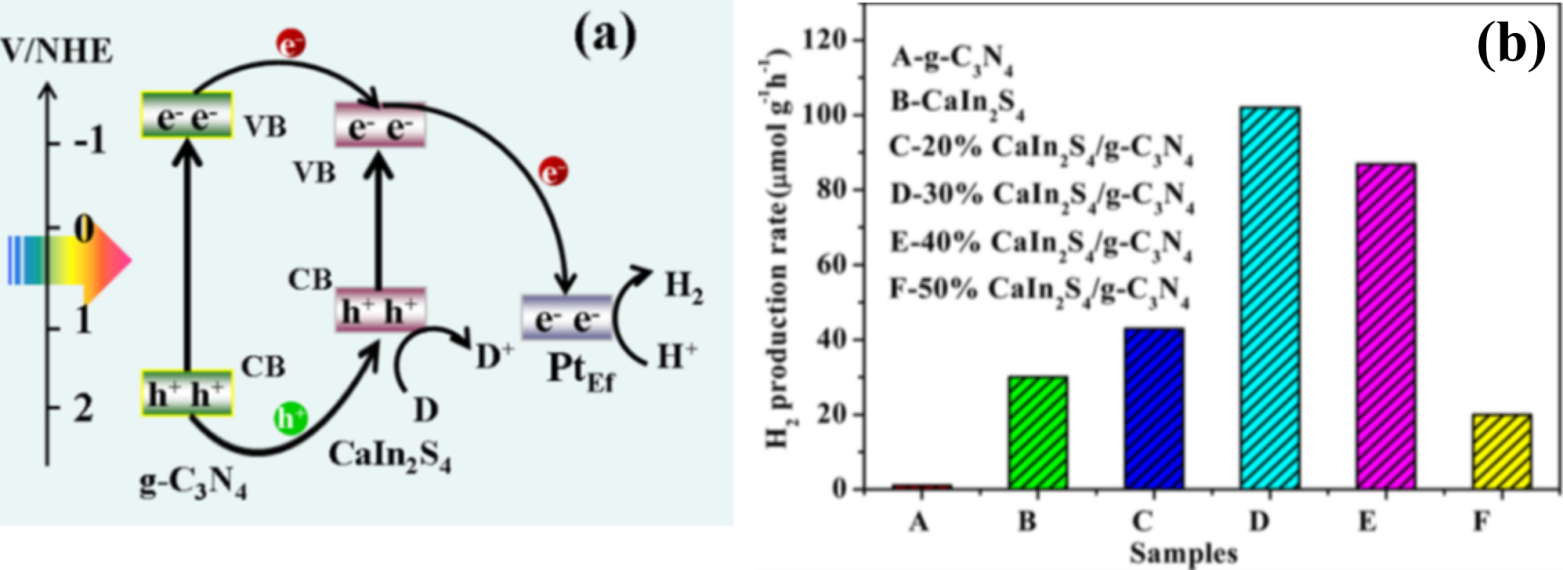
(a) Schematic illustration of the photocatalytic H_2_ production over CaIn_2_S_4_/g–C_3_N_4_ catalysts and (b) comparison of photocatalytic H_2_ production over various photocatalysts, under visible-light irradiation. Reprinted (adapted) with permission from [147], copyright 2015 American Chemical Society.

As discussed in the introduction section regarding the significance of 2D materials in photocatalytic applications, Chen et al. [147] reported a highly efficient 2D–2D heterojunction of a ternary metal sulfide CaIn_2_S_4_ with g-C_3_N_4_ nanosheets with intimate interfacial contact obtained by facile two-step hydrothermal method. The as-prepared heterojunction exhibits face-to-face contact of CaIn_2_S_4_ nanosheets with g-C_3_N_4_ nanosheets in which the interfacial contact area is very large as compared to other heterojunctions, such as point-to-line contact (OD-1D), point-to-face contact (0D-2D), line-to-line contact (1D-1D) and line-to-face contact (1D-2D). The optimized 30% CaIn_2_S_4_-g-C_3_N_4_ nanocomposite showed a H_2_ evolution rate of 102 μmol g^−1^ h^−1^, which was about 3-fold higher than pristine CaIn_2_S_4_ (Figure 11b). This enhanced H_2_ evolution was attributed to high interfacial contact between CaIn_2_S_4_ and g-C_3_N_4_ and suitable energy bands alignments, which facilitate separation of photogenerated charge carriers to reaction sites (Figure 11a). Moreover the catalyst shows excellent stability and the original phase was retained even after five reusability cycles. The H_2_ evolution mechanism was demonstrated on the basics of suitable band potentials of both the semiconductors. Under visible-light illumination, the photogenerated electron–hole formation takes place in the CB and VB of both semiconductors. As electrons transfer always takes place down potential, and holes always move up potential, the photoexcited electrons from the CB of g-C_3_N_4_ transfers to the CB of CaIn_2_S_4_, while holes from the VB of g-C_3_N_4_ also transfer to the VB of CaIn_2_S_4_. Pt serves as an excellent cocatalyst and accepts the photoexcited electrons due to its high work function, which finally reduce the H^+^ ions to generate H_2_.

#### g-C_3_N_4_-composite oxide nanocomposites

**Figure 12:**
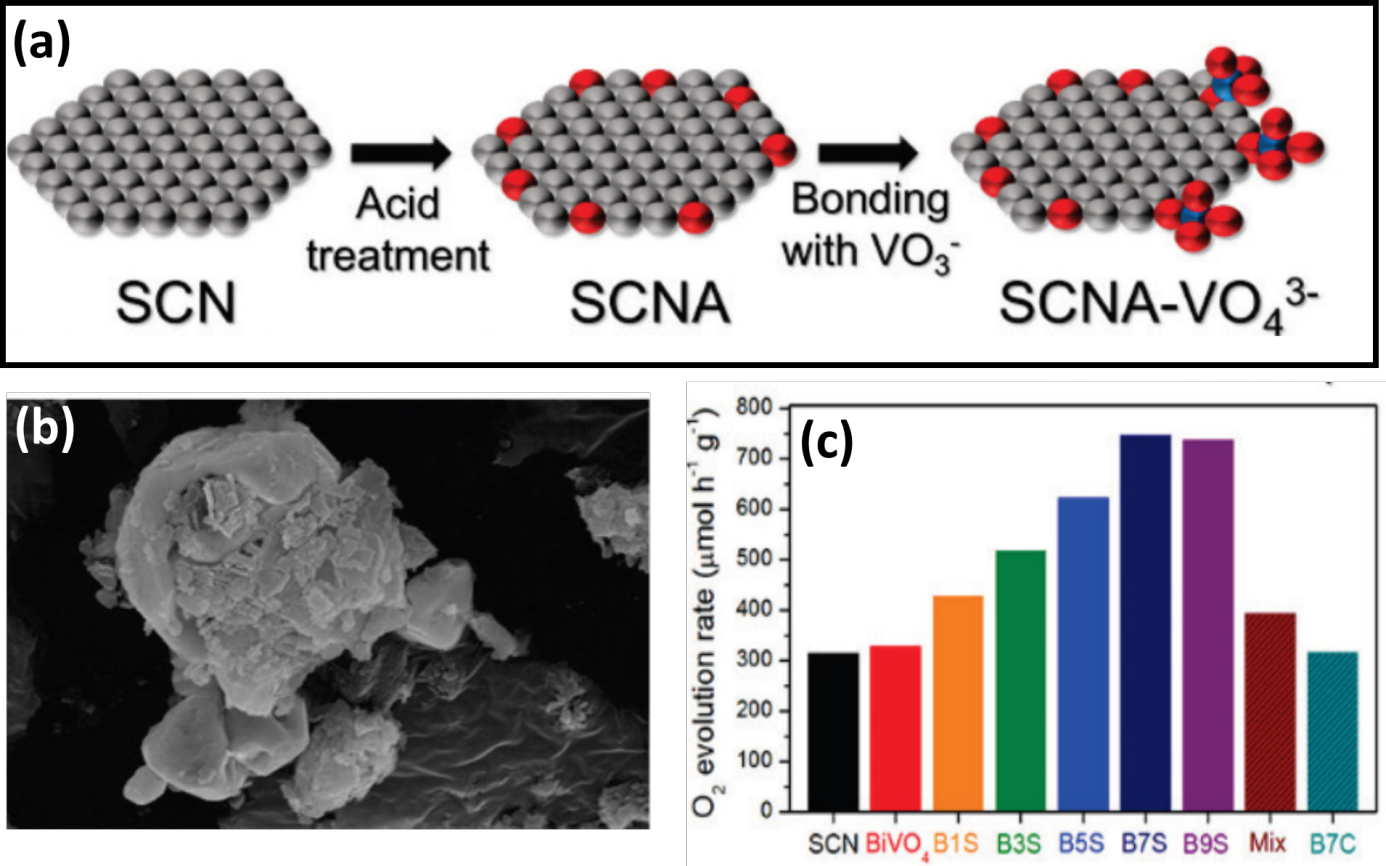
(a) Schematic diagram showing the effect of SCN acid treatment that leads to the formation of a composite between SCN and BiVO_4_ (gray for SCN, red for oxygen, and blue for vanadium atom), (b) morphology of prepared photocatalyst, and (c) photocatalytic activity of the prepared photocatalysts. Reprinted (adapted) with permission from [84], copyright 2016 American Chemical Society.

The composite oxide-based heterojunctions include very interesting nanocomposites, such as g-C_3_N_4_–Ag_3_PO_4_, g-C_3_N_4_–Ag_3_VO_4_, g-C_3_N_4_–ZnWO_4_, g-C_3_N_4_–SrTiO_3_, g-C_3_N_4_–BiWO_4_, and g-C_3_N_4_–Bi_2_WO_6_. Such kinds of nanocomposites have been widely explored with remarkably enhanced photocatalytic performance as compared to their respective bare counterparts. Recently, Woo et al. [84] reported their investigation on a sulfur-doped g-C_3_N_4_ (SCN)-BiVO_4_ nanocomposite for water oxidation reaction. Bismuth vanadate (BiVO_4_) is one of the most fascinating photocatalysts with a suitable direct band gap (2.4 eV), which is excited by visible light energy and suitably positioned CB and VB edge potentials, which are favorable for various photocatalytic reactions. However, a very high exciton recombination rate limits the photocatalytic efficiency of BiVO_4_. Hence, to overcome this issue, the heterojunction formation with an ideal material like g-C_3_N_4_ is one of the promising strategies. The sulfur-doped g-C_3_N_4_-BiVO_4_ nanocomposite was fabricated by a one-pot impregnation co-precipitation method as shown in Figure 12a. The S doping was introduced to narrow the band gap of g-C_3_N_4_ by stacking its 2p orbitals on the valence band of bare g-C_3_N_4_ which eventually contributes to increase the efficiency. Furthermore, the sulfur doping facilitates the surface oxidation of g-C_3_N_4_ during the impregnation method, and consequently, the VO_4_^3−^ tetrahedron is formed on the oxidized site of g-C_3_N_4_. A very interesting electron transfer mechanism has been discussed in the case of g-C_3_N_4_-BiVO_4_ nanocomposite in terms of a Z-scheme, wherein excited electrons from BiVO_4_ favorably combine with VB holes of g-C_3_N_4_, which is placed between the CB and VB of BiVO_4_. The high rate of O_2_ evolution (328 μmol h^−1^ g^−1^) has been achieved with an optimized g-C_3_N_4_-BiVO_4_ nanocomposite, which is 2-fold higher than pristine BiVO_4_. Figure 12 b,c presents SEM images of the g-C_3_N_4_–BiVO_4_ nanocomposite and the comparative rate of rate of O_2_ evolution for various prepared catalysts along with control samples.

It is well known that perovskite-type oxides (ABO_3_) constitute one of the promising classes of materials with diverse properties [148]. The main advantage of using the perovskite-type cubic structure is the flexibility to tune the composition of the A and B sites to form substituted materials [148]. Strontium titanate (SrTiO_3_) is an important dielectric material, with a band gap energy of 3.2 eV. The SrTiO_3_ has been explored as an ideal photocatalytic material for water splitting reactions for H_2_ fuel generation [149]. It is worth to mention here that SrTiO_3_ provides a higher potential as compared to TiO_2_ and facilitates the formation of hydrogen and oxygen. Li et al. [150] have reported the synthesis of cubic SrTiO_3_ by a polymerized complex method (solid state milling), for H_2_ evolution by water splitting under UV irradiation. It is very interesting to note that they tune the SrTiO_3_ nanoparticle size depending on the synthesis parameters. The SrTiO_3_ nanoparticles prepared by this polymerized complex route exhibit the best photocatalytic hydrogen evolution rate of 3.2 mmol h^−1^ g^−1^. This enhanced photocatalytic H_2_ evolution by water splitting of SrTiO_3_ nanoparticles could be attributed to the small particle size and hence large surface area. Small particles offer numerous active sites exposed on the surface of the catalyst. These active sites may absorb more water molecules, which are reduced by photogenerated electrons to evolve H_2_ gas. Moreover, small particles facilitate the diffusion distance from the interior to the surface of the catalyst for photogenerated charge carriers. Taking inspiration from water splitting capabilities of SrTiO_3_, various reports came on interesting nanocomposite materials based on SrTiO_3_. Subsequently, in order to enhance the photocatalytic H_2_ evolution and make SrTiO_3_ active in visible light, Irvine et al. [83] reported a unique and highly stable g-C_3_N_4_-coated SrTiO_3_ photocatalyst, which can absorb visible light for energy generation. This highly efficient photocatalyst based on g-C_3_N_4_-coated SrTiO_3_ has been synthesized in a facile manner by decomposing urea in the presence of SrTiO_3_ at 400 °C. The catalytic activity was demonstrated by photocatalytic water splitting reaction for H_2_ production and a high rate of evolution of 440 μmol h^−1^ g^−1^ has been achieved under visible light irradiation. The enhancement in photocatalytic activity could be attributed to the intimate interfacial interaction between g-C_3_N_4_ and SrTiO_3_, where photogenerated electrons and holes are effectively separated and transferred to reaction sites.

#### g-C_3_N_4_-bismuth oxyhalide nanocomposites

Recently, bismuth oxyhalides, BiOX (X = Cl, Br, I) have attracted much attention as layered materials with excellent photocatalytic properties, since the first report on the high photocatalytic activity of BiOCl in 2009 [151]. The layered structure of BiOX composed of [Bi_2_O_2_]^2+^ blocks, and the internal electric field formed in BiOX semiconductors is very effective for separation of photoexcited charge carriers to enhance the photocatalytic activity [152]. Hence, it is very interesting to couple such material with g-C_3_N_4_ to get remarkable photocatalytic enhancements. It is noteworthy to mention here that most of the p-type narrow band gap semiconductors, which have shown excellent photocatalytic activity under visible light irradiation, belong to the family of BiOX. Among them, BiOI is an attractive, p-type, visible-light responsive semiconductor due to its narrow band gap energy (1.78 eV) and is a potential to sensitize wide band gap semiconductors [153]. It is known that BiOI-based heterojunctions exhibit enhanced photocatalytic performance under visible light irradiation. Xie et al. [153] reported the synthesis of n-type porous g-C_3_N_4_ with p-type nanostructured BiOI to form a novel BiOI–g-C_3_N_4_ p–n heterojunction photocatalyst and demonstrated its efficient photocatalytic activity. The results show that the BiOI–g-C_3_N_4_ heterojunction photocatalyst exhibits superior photocatalytic activity compared to bare BiOI and g-C_3_N_4_. The visible-light photocatalytic activity enhancement of BiOI–g-C_3_N_4_ heterostructures has been attributed to the strong absorption in the visible region by both the semiconductors and improved charge transfer due to significantly suppressed recombination rate of the electron–hole pairs because of the heterojunction formed between BiOI and g-C_3_N_4_.

BiOBr is another semiconductor from the bismuth oxyhalides family that has recently gained attention in solar energy conversion due to its high photocatalytic activity and stability under UV and visible light irradiation. BiOBr is a lamellar-structured p-type semiconductor with an intrinsic indirect band gap that provides it with fast carrier mobility and prolonged electron life time [154]. However, the band gap energy of BiOBr is around 2.9 eV, indicating that it cannot absorb a significant part of visible light above 430 nm. Sun et al. [154] adopted a very interesting strategy to enhance photocatalytic activity by constructing a 2D–2D heterojunction of a BiOBr semiconductor with g-C_3_N_4_ nanosheets. This 2D–2D heterojunction exhibited enhanced photocatalytic performance due to face-to-face contact, which facilitates efficient charge transfer. They investigated the electronic coupling between the (001) plane of BiOBr and the (002) plane of g-C_3_N_4._ The favorable coupling of the crystal planes and matching band energies between BiOBr and g-C_3_N_4_ promotes the efficient transportation of photogenerated electrons and holes to reaction sites.

#### g-C_3_N_4_-noble metal nanocomposites

The noble metal nanoparticles (NPs), mainly Au, Pt, Pd, and Ru, are of great interest because of their unique electronic, optical, and magnetic properties [155]. In particular, Au NP are employed to facilitate efficient charge separation, thus serving as a Schottky barrier, wherein the charge transfer takes place from one component to another in order to align the Femi energy levels which effectively reduces the electron-hole pair recombination [155]. Moreover, the surface plasmon resonance (SPR) effect in noble metals increases the visible light utilization in nanocomposites, which leads to the improved performance [156]. Furthermore, the synthesis of nanoparticles with exposed high-energy or active facets has attracted considerable attention because they usually exhibit fascinating interfacial behaviour and have been applied in many fields including catalysis [157], sensors [158], photovoltaics [156], and energy storage applications [159]. In addition, the decoration of noble metal particles on certain substrates such as g-C_3_N_4_ is highly beneficial for enhancing the performance in many photocatalytic reactions. In particular, the use of Au NPs has proved to be extremely effective in promoting photocatalytic reactions within a wide spectral range because of size effects and the surface plasmon resonance (SPR) effect from Au NPs, leading to visible-light responsive materials. Moreover, the interfacial loading of noble metals nanoparticles on g-C_3_N_4_ could largely increase the migration of photoelectrons, which can promote the separation of electrons and holes, and thus play an important role to enhance the photocatalytic activity.

**Figure 13:**
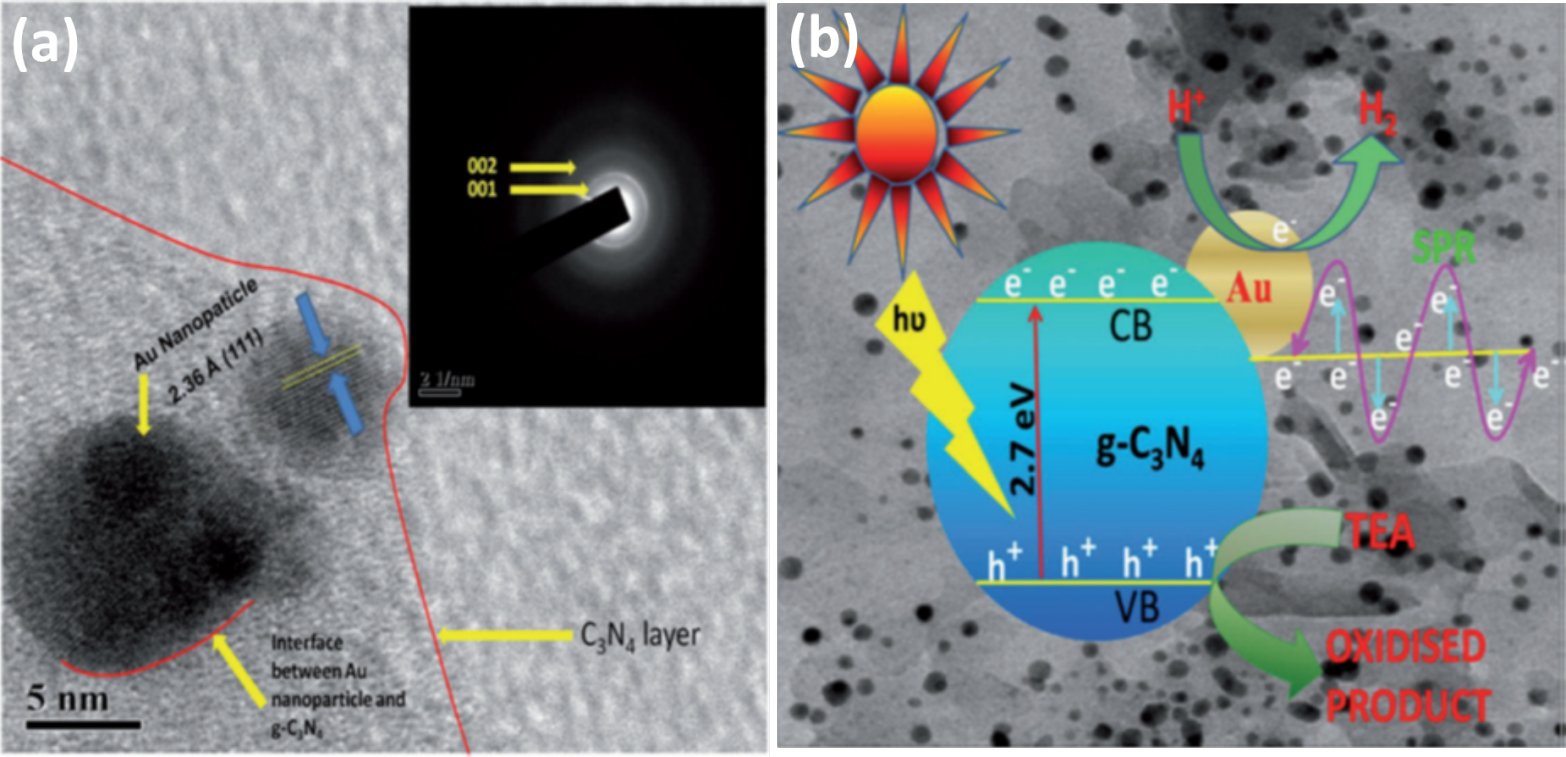
(a) HRTEM image of 1 wt % Au–g-C_3_N_4_ nanocomposite where the inset presents the corresponding SAED pattern. (b) Proposed mechanism of photocatalytic H_2_ production and SPR of Au in a Au–g-C_3_N_4_ nanocomposite. Reprinted (adapted) with permission from [160], copyright 2014 Wiley-VCH.

Parida et al. [160] explored the nanocomposite prepared by Au NP deposition on g-C_3_N_4_ by a facile deposition/precipitation method. They systematically studied the effect of Au loading on nanocomposites for visible-light-based photocatalytic H_2_ evolution. Upon exposing the nanocomposite to visible light, the electron–hole pairs are generated, resulting in the formation of a Mott–Schottky junction at the interface of the Au NP and g-C_3_N_4_ (Figure 13 a). This results in the electron transfer from the CB of g-C_3_N_4_ to the Au NP, which increases the electron density on the Au NP. Furthermore, the interaction between Au NPs and g-C_3_N_4_ results in a significant band gap reduction of g-C_3_N_4_, making it more active in visible light. The high electron density on the surface of Au NPs results in the reduction of water molecules to generate H_2_ fuel (Figure 13 b). The 1 wt % Au loaded nanocomposite was found to be the optimized composition and displayed the highest H_2_ evolution of 532 µmol, which was about 23 times higher than pure g-C_3_N_4_ along with a high photocurrent density of 49 mA cm^-2^.

Similarly, Zhu et al. reported visible-light responsive plasmonic composites of Ag@g-C_3_N_4_ having a core–shell architecture [161]. In addition to self-catalysis by noble metals, localized surface plasmon resonance (LSPR) generates local electromagnetic fields, which can be used to tune the absorption wavelength of the composites. Moreover, the core–shell composites exhibit 3D contact between the metal core and semiconductor shell, which highly facilitates the plasmonic energy transfer process. This also provides stability by preventing metals from corrosion and aggregation. These Ag@g-C_3_N_4_ core–shell composites have shown excellent activity for H_2_ evolution by water splitting under visible light irradiation. The photoluminescence (PL) emission spectra of Ag@g-C_3_N_4_ core–shell composites was broadened and quenched with increasing Ag content. This is indicative of charge transfer processes from the CB of g-C_3_N_4_ to Ag and efficiently suppresses the recombination. Furthermore, the Ag@g-C_3_N_4_ material exhibits about a 4-fold higher photocurrent density than bare g-C_3_N_4_, signifying the charge separation process in the core–shell composite with a prolonged life time of the photogenerated charge species. Hence with the synergistic effect of LSPR of Ag and the facilitated charge transfer across the core–shell due to the large area interfacial contact, the optimized Ag@g-C_3_N_4_ composite exhibits about a 30-fold higher photocatalytic H_2_ evolution as compared to g-C_3_N_4_.

#### g-C_3_N_4_-other carbon-based material nanocomposites

In the past few years, the development of noble-metal-free, highly efficient photocatalysts have been the thrust area of research in scientific community as the very high cost of noble metals restricts their use on a large scale [72]. Thus research has taken a pathway towards the development of a carbon conductive support with proper electronic structure with ultrafast electron transfer and with high concentration of active sites on their surface [162]. It has been reported that graphene also acts as an excellent electron-donating modifier for g-C_3_N_4_ due to the layered structure similar to g-C_3_N_4_ and their suitable electronic, mechanical, thermal and chemical properties [162]. Thereby, combining the two related structures of carbon-based materials would integrate their respective properties together, with remarkable or unique properties in the resulting nanocomposites. For instance, graphene–g-C_3_N_4_ nanocomposites exhibit significantly improved charge transfer kinetics because of the intimate contact between graphene–g-C_3_N_4_, wherein photogenerated electron−hole transfer takes place, which eventually plays vital role in improving the photocatalytic performance. Hence for such 2D–2D nanocomposites, the enhanced photocatalytic performance could be attributed to high catalytic surface area, abundant reaction sites and formation of well-defined electron−hole puddle at the interface of the 2D materials.

**Figure 14:**
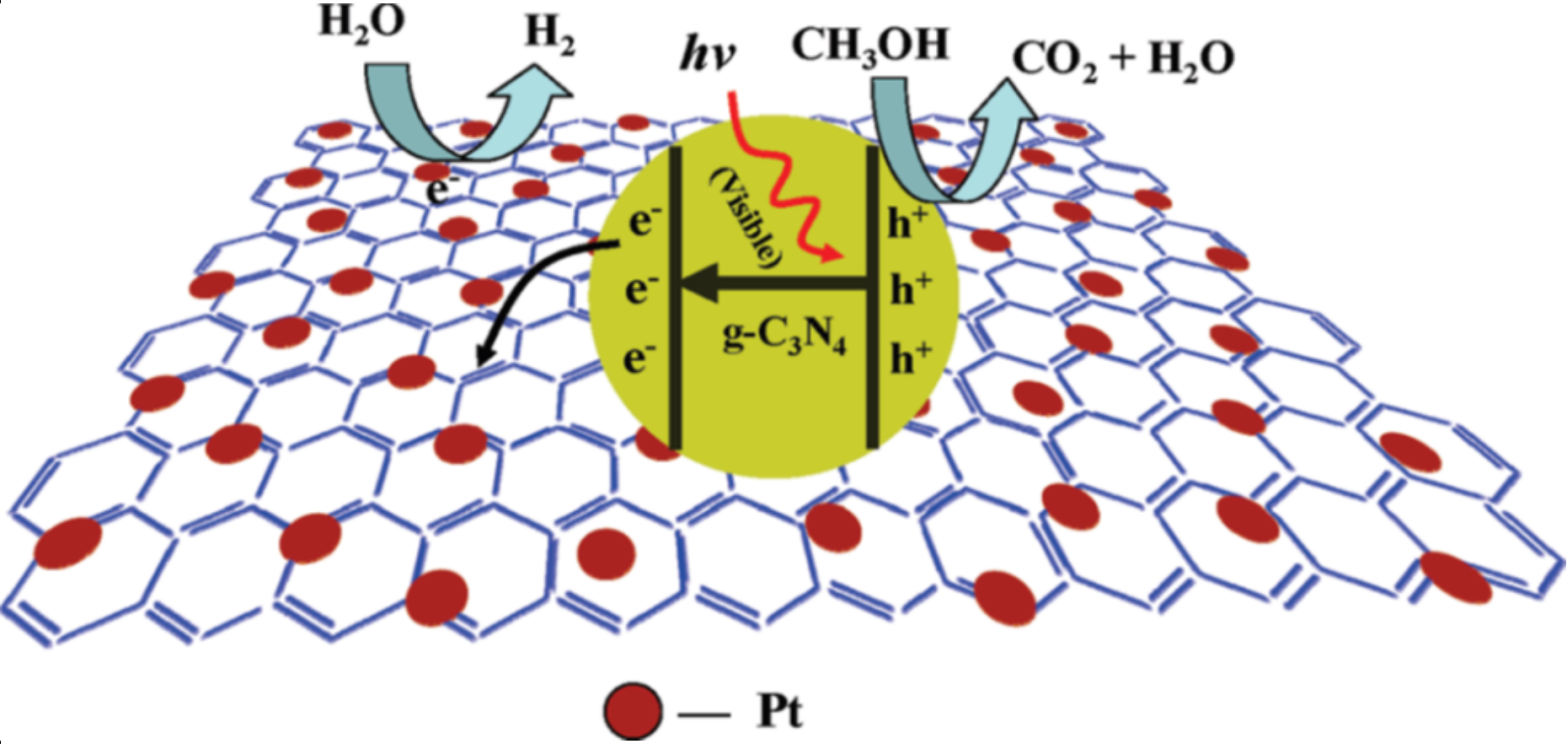
Proposed mechanism for the enhanced electron transfer in the graphene–g-C_3_N_4_ composites for photocatalytic H_2_ production under visible light irradiation. Reprinted with permission from [163] copyright 2011 American Chemical Society.

Recently, Xiang et al. reported on an intriguing nanocomposite of g-C_3_N_4_ coupled with graphene as one of the most promising metal-free visible-light active photocatalysts for H_2_ evolution [163]. The effect of graphene concentration on photocatalytic H_2_ evolution activity has been investigated and the optimum content of graphene was found to be 1 wt %. The optimized catalyst shows a H_2_ evolution rate of 451 μmol h^−1^ g ^−1^ and 2.6% apparent quantum efficiency, which was about 3-fold higher than pure g-C_3_N_4_. The reported photocatalytic mechanism for the H_2_ evolution reaction can be seen in Figure 14. It is clear that in g-C_3_N_4_ structures, N 2p orbitals constitute the VB, whereas the C 2p orbitals form the CB. Upon visible light irradiation, electrons are excited from the VB to CB of g-C_3_N_4_, which results in the formation of photogenerated electron–hole pairs. The holes from the VB are scavenged by methanol, while electrons participate in the photocatalytic reduction reaction to generate H_2_ fuel. However, the electrons are transferred from g-C_3_N_4_ to graphene sheets in the case of layered nanocomposites of graphene–g-C_3_N_4_. The transferred electrons reduce H^+^ in aqueous solution to release H_2_ as graphene acts as a conductive channel to separate the photogenerated charge carriers. The proposed photocatalytic mechanism has been further supported by photoluminescence and photocurrent studies.

In order to overcome the poor light absorption and fast recombination of charge carriers in g-C_3_N_4_, Dong et al. [164] reported a unique, metal-free, isotopic heterojunction nanocomposite with a prolonged life time of the photogenerated electrons for photocatalytic reactions by utilizing more visible light energy. They fabricated layered g-C_3_N_4_–g-C_3_N_4_ isotope heterojunctions with molecular composite precursors, urea and thiourea, which were treated under the same thermal conditions. Owing to the fact that both the precursors, urea and thiourea all possess different band structures, this gave rise to the layered g-C_3_N_4_–g-C_3_N_4_ heterojunction. Hence a facile, economic and ecologically-friendly method with earth-abundant precursors was utilized for the preparation of this isotopic heterojunction. The precursors show lattice fringes with a *d*-spacing of 0.323 nm (g-C_3_N_4_, thiourea) and 0.327 nm (g-C_3_N_4_, urea). Visible-light irradiation results in the generation of photogenerated charge carriers which tend to transfer from g-C_3_N_4_, thiourea (CN-T) to g-C_3_N_4_, urea (CN-U) driven by a CB offset of 0.10 eV, whereas the photogenerated holes transfer from CN-U to CN-T driven by a VB offset of 0.40 eV. The potential difference is considered to be the main driving force for efficient charge separation and transfer across the heterojunction. Thus the down-potential and up-potential movement of photogenerated electrons and holes, respectively, drastically reduces their recombination, which is of great significance for enhancing photocatalytic activity. Furthermore, the significance of the isotopic heterojunction was justified by photoelectrochemical (PEC) and photoluminescence (PL) studies. In the case of CN-U, a strong PL emission at 450 nm was observed, indicating the fast recombination of charge carriers, which was greatly inhibited by the heterojunction formation with CN-T. This isotopic heterojunction formation results in the redistribution of electrons on one side and holes on the other side of the heterojunction as per their band offsets. Hence intrinsic limitations have been overcome by heterojunction formation to improve quantum efficiency and construct a new class of photocatalysts materials. In addition to the works presented above, many more g-C_3_N_4_-based nanocomposites have been investigated by several researchers for photocatalytic energy generation applications. Some of the important recent reports have been summarized in Table 3.

**Table 3:** Photocatalytic H_2_ evolution over g-C_3_N_4_-based nanocomposites. LED – light emitting diode; TEOA – triethanolamine; QDs – quantum dots.

Sl no.	photocatalyst	synthesis route	light source	sacrificial agent	H_2_ production	ref. (year)

1	g-C_3_N_4_–SrTiO_3_	co-precipitation l and calcination	250 W UV–vis lamp	–	440 µmol h^–1^·g^–1^	[83] (2011)
2	g-C_3_N_4_–SrTiO3:Rh	solid state reaction	300 W Xe lamp	methanol	223.3 µmol·h^–1^	[165] (2012)
3	g-C_3_N_4_–NiS	hydrothermal	visible light	TEOA	48.2 µmol·h^–1^	[166] (2013)
4	g-C_3_N_4_–MoS_2_	impregnation	visible light	lactic acid	20.6 µmol·h^–1^	[167] (2013)
5	g-C_3_N_4_–CdS	solvothermal and chemisorption	350 W Xe arc lamp	–	4152 µmol h^–1^·g^–1^	[168] (2013)
6	g-C_3_N_4_–Cu_2_O	reduction	300W Xe lamp	TEOA	241.3 mol h^–1^·g^–1^	[169] (2014)
7	g-C_3_N_4_–SnO_2_	chemical synthesis	300W Xe lamp	TEOA	900 µmol h^–1^·g^–1^	[170] (2014)
8	g-C_3_N_4_–N-TiO_2_	electrospinning	300 W Xe arc lamp	methanol	8931.3 μmol·h^–1^·g^–1^	[171] (2015)
9	g-C_3_N_4_–C-N-TiO_2_	solvothermal	300 W Xe lamp	TEOA	39.18 µmol h^–1^·g^–1^	[172] (2015)
10	g-C_3_N_4_–CdS QD	thermal polymerization	300W Xe lamp	TEOA	601 µmol·h^−1^	[173] (2015)
11	g-C_3_N_4_–Au–CdS	in situ reduction and photodeposition	visible light	TEOA	277 µmol·h^−1^	[174] (2015)
12	g-C_3_N_4_–N-CeOx	annealing	300 W Xe lamp	TEOA	292.5 µmol· h^–1^·g^–1^	[175] (2015)
13	g-C_3_N_4_–MgFe_2_O_4_	sol−gel and auto combustion	300 W Xe lamp	TEOA	30.09 μmol·h^−1^	[176] (2015)
14	g-C_3_N_4_–InVO_4_	hydrothermal	300 W Xe arc lamp	methanol	212 µmol·h^–1^·g^–1^	[146] (2015)
15	g-C_3_N_4_–TiO_2_	solvothermal	UV LED (3 W, 420 nm)	methanol	5.6 µmol·h^−1^	[177] (2016)
16	g-C_3_N_4_–TiO_2_	calcination and solvothermal	AM1.5 solar power system	methanol	186.9 μmol·h^−1^	[178] (2016)
17	g-C_3_N_4_–Ni@NiO-CdS	reduction	300 W Xe lamp	TEOA	1258.7 μmol·h^−1^·g^−1^	[179] (2016)
18	g-C_3_N_4_@TiO_2_–CdS	hydrothermal	UV LED (3 W, 420 nm)	–	75.2 µmol·h^−1^	[180] (2017)
19	g-C_3_N_4_–Ca_2_Nb_2_TaO_10_	thermal condensation and polymerization	300 W Xe arc lamp	TEOA	43.54 µmol·h^−1^	[181] (2017)

#### Photocatalysts for environmental remediation applications

Over the years, it has been observed that substantial research efforts have been devoted to the design and development of functional nanomaterials, which can utilize maximum light energy and remove various kinds of organic and inorganic pollutants from water. It has been noticed that most of these pollutants cannot be removed completely by biological or conventional treatment methods because of their high chemical stability or strong resistance to mineralization [182]. As environmental pollution, and especially water contamination, has surpassed the threshold of the natural purification process due to rapid industrialization, there is an urgent need to develop low cost, environmentally benign methods, which can effectively remove pollutants from contaminated water. The chemical oxidation of pollutant dyes, such as methylene blue (MB), methyl orange (MO), rhodamine B (RhB) can lead to their complete mineralization [183]. This oxidation process involves the in situ generation of highly reactive oxidative species, such as hydroxyl radicals (^*^OH), superoxide radicals (O_2_^−*^) and holes (h^+^) during photocatalytic reaction [12]. These highly oxidative species react with target molecules (pollutants) and bring about their complete mineralization. The heterogeneous photocatalysis has turned out to be one of the most appealing options for pollutant removal due to its potential to mineralize pollutants by utilizing the solar energy spectrum [12]. Carbon-based 2D materials (mainly graphene and g-C_3_N_4_) have been extensively employed as nanocomposites because of their high specific surface area, which can adsorb large quantities of pollutants. Therefore, more adsorption of pollutants over the catalyst surface is one of the crucial parameters in addition to a low recombination rate and fast charge transfer to generate active oxidative species during oxidative degradation processes.

Generally, the photocatalytic degradation mechanism over semiconductor-based nanocomposites can be summarized as the following [12]:


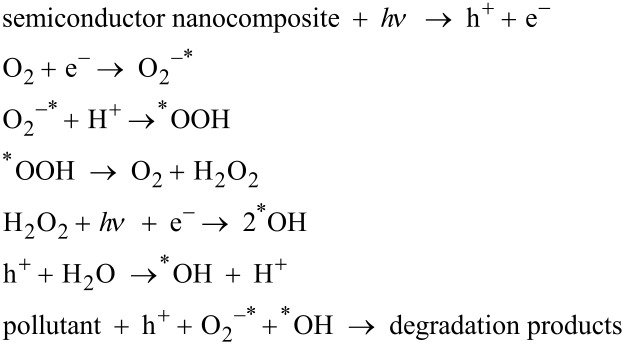


It is clear from the above reactions that when a photocatalyst is subjected to light, electron–hole pair formation takes place after absorption of photon energy (*h*ν) is equal to or greater than the band gap of the semiconductor. Then, photogenerated electrons in the CB of the semiconductor and the holes in its VB migrate to the surface of the semiconductor photocatalyst. This is followed by the in situ generation of active oxidative species, hydroxyl radicals (^*^OH), superoxide radicals (O_2_^−*^) and holes (h^+^). The ^*^OH radicals are primary oxidative species in photocatalytic reactions to degrade pollutants, which are formed in aqueous solution by two routes. Initially, water is photo-oxidized by h^+^ followed by reaction of O_2_^−*^ with protons (H^+^) to form ^*^OOH, which in turn generates O_2_ and H_2_O_2_ that finally decomposes to form ^*^OH. Furthermore, the photogenerated h^+^ also have the tendency to degrade organic pollutants directly depending on oxidative conditions. In order to increase the efficiency of photocatalytic reactions, the use of hole scavengers is always preferred, which effectively suppresses the photogenerated carrier recombination.

#### Graphene-based binary nanocomposites for environmental remediation

As explained in a previous section, the extraordinary optical and electrical properties of graphene makes it a perfect material for various practical applications. It is anticipated that bulk graphene can preserve its extraordinary properties. However, the strong van der Waals interactions result in restacking of graphene sheets and its conductivity is partly revived after reduction from graphene oxide to the reduced form which diminishes its accessible surface area [184]. Extensive studies have been devoted to tackle this problem. One of the effective ways is nanocomposite formation with metal sulfide/oxide semiconductors, noble metals etc., which can effectively avoid re-stacking of individual graphene sheets. This retains the high conductivity and high specific surface area availability for practical applications such as photocatalytic pollutant removal.

Graphene–semiconductor-based binary nanocomposites with excellent visible-light response have been explored widely for pollutant degradation because of their extraordinary performance. Based on the visible-light response, the narrow band gap semiconductors, mainly MoS_2_ (*E*_g_ = 1.86 eV) in nanocomposite with graphene, have been intensively studied. Pan et al. [185] reported binary nanocomposites of MoS_2_–reduced graphene oxide prepared by a microwave-assisted method. The graphene oxide was reduced to RGO with MoS_2_ precursor thioacetamide solution during microwave treatment. This binary nanocomposite was tested for visible-light-based photocatalytic degradation of MB as a model pollutant. The results indicate about 99% degradation occurred within 60 min of visible-light irradiation for nanocomposites optimized at 0.5 wt % RGO in the photocatalyst. This enhanced photocatalytic performance has been attributed to excellent dye adsorption on RGO and improved charge transfer between MoS_2_ and RGO. Subsequently, Chen et al. also reported similar binary MoS_2_–graphene oxide (GO) nanocomposites by hydrothermal method for solar-light-based degradation of MB. The MoS_2_ content was systematically varied in the nanocomposites and composition where 10 wt % of MoS_2_ proved to be best composition for enhanced photocatalytic performance for MB removal [186].

Furthermore, visible-light responsive catalysts, such as CdS, have been explored by Wang et al. [187] who reported visible-light active CdS–graphene nanocomposites prepared by hydrothermal methods for dye degradation. Interestingly, the loading of graphene onto CdS further decreases the band gap of CdS, which signifies the strong interaction between both the components in binary nanocomposites and has been supported by the diffuse reflectance UV–vis spectroscopy. Moreover, the transient photocurrent response studies further confirm the CdS–graphene heterojunction formation and excellent photogenerated charge separation, which leads to more 95% degradation of MO in only 60 min of irradiation.

Besides acting as an excellent electron acceptor/transporter, the role of graphene as a photosensitizer has also been reported. Zhu et al. [188] have reported the ZnWO_4_–graphene nanocomposite and the photocatalytic activity was demonstrated both under UV and visible light for MB degradation. The visible-light responsive activity of ZnWO_4_–graphene nanocomposites was about 7-fold higher than bare ZnWO_4_, which could be ascribed to the generation of ^*^OH and O_2_^−*^ because of charge transfer from graphene (LUMO) to the CB of ZnWO_4_. The transferred electrons in the CB of ZnWO_4_ reduce the dissolved O_2_ to generate O_2_^−*^. This explains the photosensitizer role of graphene in which photogenerated electron–hole pair formation by promotion of electrons from HOMO to LUMO. In addition to this, ZnWO_4_ is UV-excited as per its band gap energy (3.08 eV), which also results in photogenerated charge carrier formation. However, this work does not exclude the possibility of dye sensitization which could lead to fast charge transfer and enhanced photocatalytic activity. There are many reports available [189–190], which explain the significance of dye sensitization to enhance photocatalytic activity.

**Figure 15:**
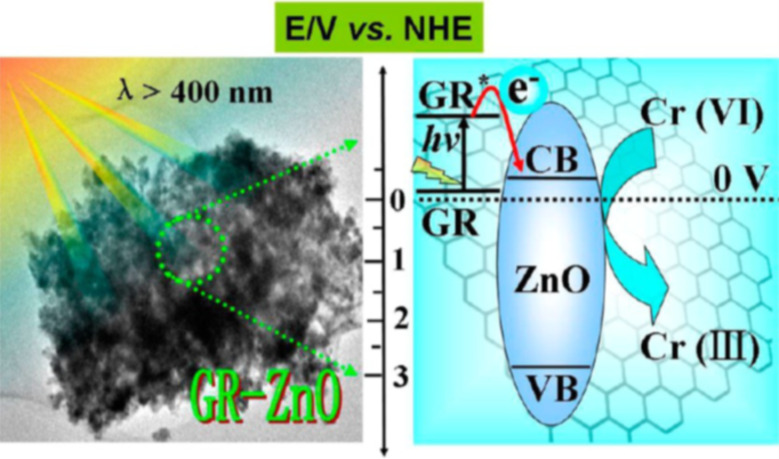
Schematic illustration depicting the photosensitizer role of graphene in GR–ZnO nanocomposites for the photocatalytic reduction of Cr(VI) in aqueous solution. Reprinted with permission from [128], copyright 2013 American Chemical Society.

Thus, to avoid self-induced photosensitization of the reaction substrate, Xu et al. reported graphene–ZnO-based nanocomposites with strong interfacial bonding by in situ growth of graphene (GR) sheets on ZnO [128]. This nanocomposite has been utilized for photocatalytic reduction of Cr(VI) to Cr(III) in aqueous solution under visible-light irradiation. The band gap of ZnO is about 3.37 eV, hence it cannot absorb visible light and this excludes the possibility of photocatalytic activity because of ZnO excitation. Thus upon visible-light irradiation, electron promotion from HOMO (GR) to LUMO (GR) takes place, from where photogenerated electrons are transferred to the CB of ZnO and further participate in the reduction reaction as presented in Figure 15.

#### Graphene-based ternary nanocomposites for environmental remediation

In addition to the binary nanocomposites, graphene-based ternary nanocomposites have also attracted much attention for environmental remediation applications. In order to further improve the photocatalytic performance, heterojunction construction of ternary nanocomposites with suitable energy band alignments have been explored. Such ternary heterojunctions could benefit the charge transfer across the interface as per suitable band potentials to facilitate the separation of photogenerated charge carriers efficiently. In addition to the improved charge transfer kinetics, ternary nanocomposites showed excellent light absorption owing to the presence of a three-component system, which can better utilize a wide range of the solar energy spectrum in comparison to binary nanocomposites. Recently, our group reported the synergetic effect of MoS_2_–RGO doping of ZnO nanoparticles to enhance its photocatalytic performance for pollutant removal [191]. The ZnO–MoS_2_–RGO ternary nanocomposites were prepared by a facile hydrothermal method with varying content of MoS_2_–RGO nanosheets. Firstly, MoS_2_–RGO nanosheets were prepared and then its suspension with zinc acetate dihydrate, Zn(CH_3_COO)_2_·2H_2_O, sodium hydroxide (NaOH) was made and treated hydrothermally to afford the final ternary nanocomposites exhibiting intimate contact between ZnO–MoS_2_–RGO. The photocatalytic activity of the prepared ternary nanocomposites was examined by the studying the degradation of a coloured pollutant, MB dye, and a colourless pollutant, carbendazim, a hazardous fungicide under natural sunlight irradiation. The enhanced photocatalytic activity of as-prepared ternary nanocomposites, as compared to bare ZnO nanoparticles, has been attributed to the synergetic effect between MoS_2_–RGO. The charge transfer occurs as per the CB and VB potentials of ZnO and MoS_2_. The CB of ZnO (−0.31 eV vs NHE) is more negative than that of MoS_2_ (−0.13 eV vs NHE), which favours the photogenerated electron transfer from the CB of ZnO to the CB of MoS_2_. Furthermore, MoS_2_ has a more negative CB than RGO, which has Fermi level at −0.08 eV vs NHE, facilitating the charge transfer to RGO. These transferred electrons form reactive oxidative species ^*^OH, which degrade both the coloured and colourless pollutants during the photocatalytic process. The high surface area of MoS_2_–RGO nanosheets adsorb pollutants effectively thereby contributing to their efficient degradation.

To further prove the role of graphene as an excellent electron accepting/shuttling system with high pollutant adsorption ability, our group reported another ternary nanocomposite composed of CdS–ZnO–RGO for degradation of MO under visible light and natural sunlight irradiation [192]. More than 90% of the dye was removed from water in 60 min under natural sunlight irradiation, while it took about 90 min under visible-light irradiation. Under natural sunlight irradiation, both of the semiconductors (CdS and ZnO) are photoexcited and charge transfer takes place from the more negative CB of CdS (−0.66 eV vs NHE) to the CB of ZnO (−0.31 eV vs NHE). The photogenerated electrons from the CB of ZnO are readily transferred to RGO because of the high work function value of ZnO (5.2–5.3 eV) as compared to RGO (4.5 eV). Simultaneously, the transfer of the holes takes place up-potential from the VB of ZnO to the VB of CdS. As the Fermi level of RGO is −0.08 eV vs NHE, which is more positive than the redox potential of O_2_/O_2_^−*^ (−0.13 V), O_2_^−*^ cannot be formed but H_2_O_2_ formation was favoured as per its redox potential (O_2_/H_2_O_2_ = +0.695 eV vs NHE). This H_2_O_2_ further decomposed to form ^*^OH. Thus photogenerated electron–hole pairs are effectively separated, which improves the efficiency of the reaction. Holes along with ^*^OH resulted in the degradation of adsorbed MO on the photocatalyst surface.

**Figure 16:**
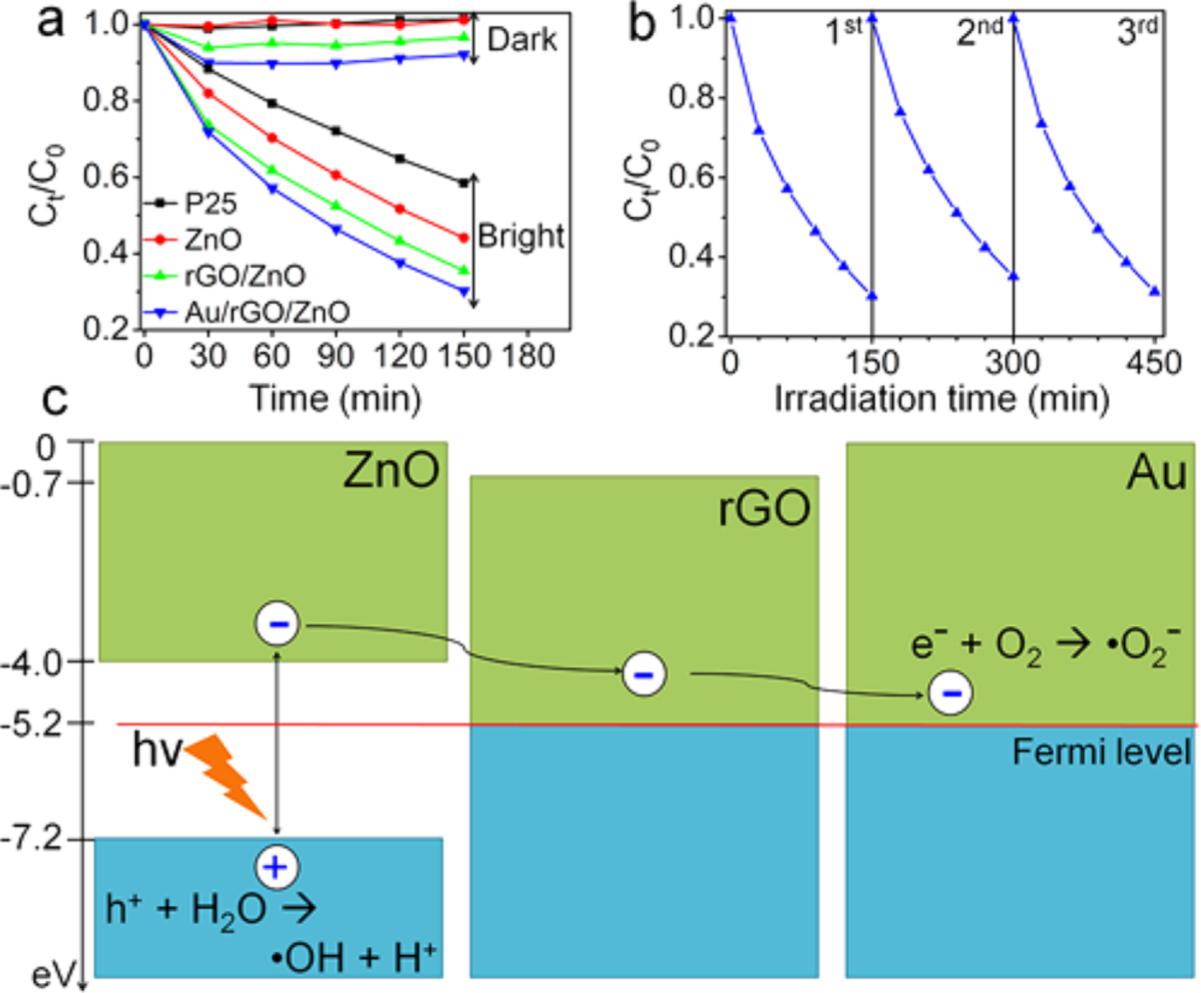
(a) Diagram showing the superior photocatalytic activity of the Au–RGO–ZnO heterostructures, (b) reusability cycles of Au–RGO–ZnO, and (c) a schematic illustration of the charge transfer in Au–RGO–ZnO heterostructures. Reprinted with permission from [119], copyright 2015 American Chemical Society.

Many research groups have utilized the SPR effect of noble metals like Au and Ag to utilize the visible region of the solar energy spectrum by the formation of a Schottky barrier for facile charge transfer to fabricate ternary nanocomposites with promising photocatalytic activity. Hahn et al. [119] fabricated Au NP-decorated, reduced graphene oxide (RGO)-wrapped, ZnO hollow spheres. The unique structure of the ZnO hollow spheres provided a very high charge transfer of around 87 ps, which is better than other nanostructures like nanorods (128 ps), nanoparticles (150 ps), etc. Au-decorated heterostructures showed an improved charge transfer efficiency of 68% as compared to their binary counterpart (RGO–ZnO) at only 40.3%. These high charge transfer kinetics resulted in improved photocatalytic activity of nanocomposites towards MB degradation as can be seen from Figure 16a,b. In addition, the high surface area of the Au–RGO–ZnO heterostructures (28.9 m^2^g^−1^), as compared to RGO–ZnO (17.9 m^2^g^−1^) and ZnO (12.7 m^2^g^−1^) resulted in excellent adsorption of MB, which is readily degraded. The photocatalytic degradation mechanism of the Au–RGO–ZnO nanocomposite is presented in Figure 16c. Upon UV light irradiation, electron−hole pairs are generated in the ZnO. The photogenerated electrons from the CB of ZnO are transferred to RGO due to the suitable work function value of RGO (4.5 eV) as compared to 5.2–5.3 eV for ZnO and 5.1 eV for Au nanoparticles. These transferred photogenerated electrons react with dissolved O_2_ to form O_2_^•−^ while photogenerated holes can generate ^*^OH by reacting with water. These oxidative reactive species finally result in the mineralization of pollutants.

In addition to these binary and ternary graphene-based nanocomposites, there are many reports available in literature on the use of other graphene-based nanocomposites for environmental remediation application. Some of the noteworthy recent reports have been summarized in Table 4.

**Table 4:** Graphene-based nanocomposites for environmental remediation. MCM 41 – mesoporous silica; H_2_TCPP – *meso*-tetrakis(4-carboxyphenyl)porphyrin; RGO – reduced graphene oxide; GR – graphene; EE2 – 17α-ethinylestradiol.

Sl no.	photocatalyst	light source	pollutant (concentration)	*t*_completion_ (min)	ref. (Year)

1	GO–TiO_2_	visible light 1000 W Xe lamp	MO (12 mgL^−1^)	180 min	[193] (2010)
2	GR–TiO_2_–MCM41	100 W Hg lamp	2-propanol (2.6 mmol L^−1^)	–	[194] (2010)
3	RGO–ZnO	UV light	MB (5 mg L^−1^)	260 min	[195] (2011)
4	GO–Ag–AgX (X = Cl, Br)	500 W Xe arc lamp	MO (15 mg L^−1^)	40 min	[196] (2011)
5	GR–ZnFe_2_O_4_	500 W Xe lamp	MB (20 mg L^−1^)	90 min	[197] (2011)
6	GR–TiO_2_	150 W high-pressure Xe lamp	MB (1 mg · L^−1^)	180 min	[198] (2011)
7	GR–InNbO_4_	500 W Xe lamp	MB (5 mg L^−1^)	90 min	[199] (2011)
8	GR–Bi_2_WO_6_	500 W Xe lamp	RhB (10^−5^ M)	16 min	[200] (2011)
9	GR–TiO_2_	UV light, mercury lamp solar light	RhB (0.5 × 10^−5^ M)	40 min	[201] (2012)
10	RGO–SnO_2_	350 W Xe lamp	MB (2.7 × 10^−5^ M)	360 min	[202] (2012)
11	RGO@ZnO	simulated solar light	RhB	120 min	[48] (2012)
12	RGO–ZnO	12 W UV lamp	MB (5.0 × 10^−5^ M)	130 min	[203] (2012)
13	GR–TiO_2_	UV light, 40 W cylindrical black light bulb	MB (0.01 mM)	–	[204] (2012)
14	GR–Fe^3+^–TiO_2_	UV–vis light	MB (4.5 ppm), formaldehyde (3000 ppmV)	150 min, 90 min	[205] (2013)
15	RGO–SnS_2_	500 W Xe lamp	Rh B (10 mg L^−1^), phenol (10 mg L^−1^)	120 min, ≈240 min	[206] (2013)
16	RGO–MoS_2_	visible light, 5 W white LED	MB (60 mg L^−1^)	60 min	[185] (2014)
17	GR–TiO_2_	UV light, 100 W mercury lamp	MO (10^-4^ mol L^−1^ )	240 min	[207] (2014)
18	GR–CaTiO_3_	15 W low-pressure mercury lamp	MO (1 mg L^−1^)	60 min	[208] (2014)
19	RGO–TiO_2_–ZnO	300 W Xe lamp	MB (0.3 mg L^−1^)	120 min	[209] (2015)
20	RGO–KTaO_3_	visible light	phenol (0.21 mM)	60 min	[210] (2015)
21	RGO–H_2_TCPP–TNT	halogen lamp	MB (10 mg L^−1^)	120 min	[211] (2016)
22	RGO–Pt–TiO_2_	300 W Xe lamp irradiation	nitrobenzene (0.01M)	480 min	[212] (2016)
23	RGO–Ag–Bi_2_MoO_6_	300 W halogen tungsten lamp	phenol (10 mg L^−1^)	300 min	[213] (2016)
24	RGO–Ag–ZnFe_2_O_4_	300 W Xe lamp	EE2 (2.0 mg L^−1^)	240 min	[214] (2016)
25	RGO–Pd–Bi_2_MoO_6_	300 W halogen tungsten lamp	phenol (10 mg L^−1^)	300 min	[215] (2017)

#### g-C_3_N_4_-based nanocomposites for environmental remediation

g-C_3_N_4_ is an important material of interest for environmental remediation applications in the form of nanocomposites [95,216]. The structure of g-C_3_N_4_ is composed mainly of C–N bonds, which makes it a mildly basic catalytic material. Furthermore, the replacement of C by N in the six-membered ring leads to more basicity, which is beneficial for reactions like the nitrogen monoxide (NO) decomposition [217]. NO is a hazardous pollutant that causes various environmental issues such as acid rain, photochemical smog, etc. The direct decomposition reaction of NO into N_2_ and O_2_ is not feasible due to various issues in real conditions [218]. Moreover, atmospheric O_2_ prevents adsorption of NO on active sites of the catalyst surface and hence decreases the activity. The basic groups on the surface of g-C_3_N_4_ provide resistance to O_2_, the polar groups C–N–C favours adsorption of NO on its surface. Therefore g-C_3_N_4_ is an ideal catalyst for the NO decomposition reaction [217].

Recently, Zhang et al. [217] reported n–n type nanocomposites of CeO_2_–g-C_3_N_4_ by an in situ pyrolysis method with enhanced photocatalytic activity for phenol and NO removal under visible-light irradiation. The optimized CeO_2_–g-C_3_N_4_ catalyst with 8% CeO_2_ in the nanocomposite shows the best photocatalytic performance. The photocatalysts having more CeO_2_ content show decreased activity due to agglomeration of CeO_2_ over g-C_3_N_4_ nanosheets, which can destruct interfacial contact and hence the charge transfer across it. This optimized 8% CeO_2_–g-C_3_N_4_ photocatalyst exhibited a high photocurrent (0.35 µA) as compared to bare CeO_2_ (0.06 µA) and g-C_3_N_4_ (0.14 µA), which clearly signify the high interfacial charge separation and suppressed recombination rate of the photogenerated charge carriers. The CB potential of g-C_3_N_4_ (−1.09 eV) is more negative as compared to CeO_2_ (−0.79 eV) which favours the photogenerated electron transfer down-potential to the CB of CeO_2_ from the CB of g-C_3_N_4_. This is followed by hole transfer from the VB of CeO_2_ to the VB of g-C_3_N_4_. Hence photogenerated charge species are effectively separated from each other at intimate interfacial contact between CeO_2_ and g-C_3_N_4_. The density of holes increases in the VB of g-C_3_N_4_, which causes the mineralization of pollutants because of its strong oxidizing power. On the other hand, electrons from the CB of CeO_2_ react with the dissolved O_2_ to form O_2_^−*^, contributing to the degradation of the pollutants.

**Figure 17:**
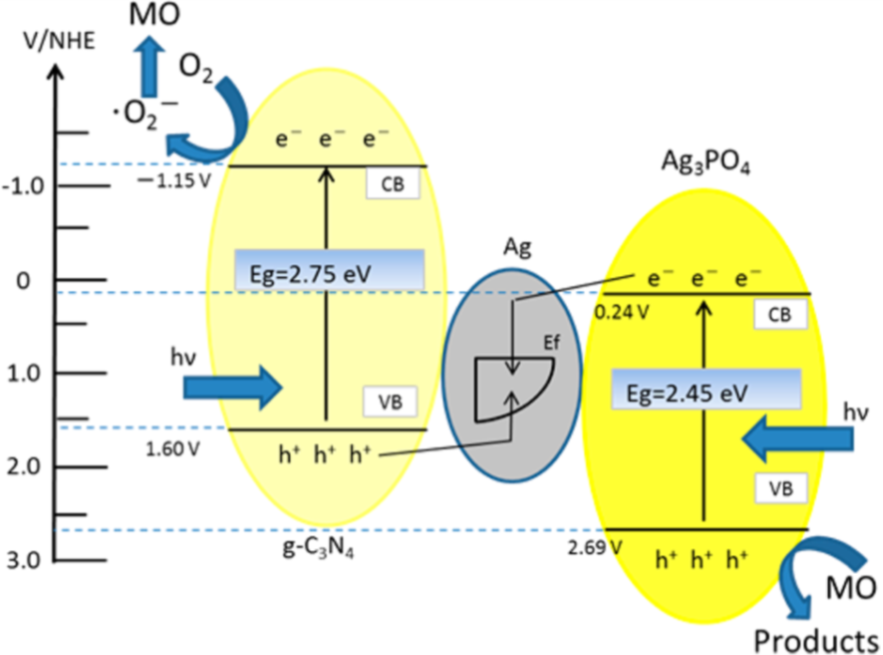
Z-scheme photocatalytic mechanism of the g-C_3_N_4_–Ag_3_PO_4_ hybrid photocatalyst under visible-light irradiation (>440 nm). Reprinted with permission from [219], copyright 2014 American Chemical Society.

A highly efficient g-C_3_N_4_–Ag_3_PO_4_ nanocomposite for MO removal under visible light was reported by Katsumata et al. [219]. Ag_3_PO_4_ is one of the more interesting semiconductors with a 2.45 eV band gap and high oxidative power for pollutant degradation. The in situ precipitation method was employed for g-C_3_N_4_–Ag_3_PO_4_ nanocomposite synthesis, during which Ag nanoparticle formation on the surface of catalysts plays a crucial role in photocatalytic activity. The charge transfer in this nanocomposite takes pace through the Z-scheme process. As is clear from Figure 17, visible-light irradiation results in the formation of photogenerated electrons in the CB and holes in the VB of both the semiconductors. The photogenerated electrons from the CB of Ag_3_PO_4_ migrate to the Ag nanoparticles through the Schottky barrier due to the more positive Fermi level of Ag. Moreover, the Fermi level of Ag is more negative than the VB potential of g-C_3_N_4_, which leads to the hole migration from the VB of g-C_3_N_4_ to Ag. Hence Ag nanoparticles at the interface of g-C_3_N_4_–Ag_3_PO_4_ acts as a charge separator and oxidative species are formed by CB electrons in g-C_3_N_4_, which brings about pollutant degradation. Holes from the VB of Ag_3_PO_4_ itself oxidize the pollutants. The g-C_3_N_4_–Ag_3_PO_4_ nanocomposite was able to degrade MO dye in just 5 min of irradiation, which illustrates the high efficiency of the photocatalyst. This excellent photocatalytic activity could be attributed to the efficient photogenerated charge separation by the Z-scheme process, wherein Ag nanoparticles as charge separation centers leads to fast charge transfer across interface in g-C_3_N_4_–Ag_3_PO_4_ nanocomposites.

Zhao et al. [220] reported a very interesting study on the band gap tuning of g-C_3_N_4_-RGO nanocomposites. They reported that by controlling the reduced graphene oxide (RGO) content in the nanocomposite, its band gap can be easily tuned. The g-C_3_N_4_–RGO nanocomposite exhibits a considerably reduced band gap as compared to bare g-C_3_N_4_. It was very interesting that an optimized RGO content in the nanocomposite led to a positive shift of the VB edge potential, thereby increasing its oxidation power. The decrease in the band gap energy of the nanocomposite was ascribed to the covalent bond formation of C–O–C between g-C_3_N_4_ and RGO, which has been confirmed by Fourier transform infrared (FTIR) and X-ray photoelectron spectroscopy (XPS). This nanocomposite exhibits improved photocatalytic activity for the degradation of rhodamine B and 4-nitrophenol under visible light irradiation, which could be attributed to the enhanced visible light absorption by band gap narrowing, high oxidation power and the excellent electron conductivity across the g-C_3_N_4_–RGO interface. Many g-C_3_N_4_ nanocomposites based on heterojunction formation with graphene have been reported with high quantum efficiency for various photocatalytic applications.

Huang et al. [221] reported a novel ternary nanocomposite composed of g-C_3_N_4_–Ag–TiO_2_ with enhanced photocatalytic activity for pollutant removal. The Ag was photodeposited as an interlayer between g-C_3_N_4_ and TiO_2_ and this ternary nanocomposite was demonstrated for visible-light-based photocatalytic activity. The visible-light response of the nanocomposite was enhanced due to the SPR effect of Ag and the interface formation between Ag–g-C_3_N_4_–TiO_2_. The photocatalytic mechanism has been discussed on the basis of CB and VB edge potentials in this ternary g-C_3_N_4_–Ag–TiO_2_ nanocomposite. The CB and VB edge potentials of g-C_3_N_4_ were at −1.23 and +1.52 eV, while those of TiO_2_ were at −0.30 and +2.92 eV, respectively. Under visible-light irradiation, only g-C_3_N_4_ was excited because of its suitable band gap. The photoexcited electron transferred to the CB of TiO_2_ because of the more negative CB potential of g-C_3_N_4_. Furthermore, Ag NPs in the interlayer of the two semiconductors played a crucial role as an electron-conduction bridge. Moreover the Schottky barrier formation takes place at the interface of the Ag and TiO_2_ nanoparticles, which facilitates this electron transfer in addition to enhanced visible-light response due to its SPR effect.

The nanocomposites of g-C_3_N_4_ with other cabon-based materials, such as graphene, have been investigated thoroughly as efficient, low cost and metal-free photocatalysts for removal of various pollutants. The development of such nanocomposites is generally based on some nanoparticle/nanorod/nanosheet heterostructure, which are nowadays a very common strategy explored on a large scale. In this regard, recently, Jiang et al. [222] explored a very effective 3D porous aerogel based on g-C_3_N_4_ and GO nanosheets for photocatalytic environmental remediation. This aerogel was prepared by the hydrothermal co-assembly method and utilized for MO dye removal under visible-light irradiation. The GO nanosheets with porous structure and high pollutant adsorption capability were utilized for nanocomposite formation with g-C_3_N_4_ . The main advantage of the 3D porous structure is that it can inhibit the stacking of nanosheets and make more active sites available for catalytic reaction. The g-C_3_N_4_ acts as a photocatalyst and electron–hole pairs are generated by visible light absorption. GO makes a 3D porous structure and facilitates the charge transfer process at the large coherent interface to generate reactive oxidative species, which can mineralize the MO dye effectively. More than 90% of MO was removed by a porous aerogel of g-C_3_N_4_ and GO nanosheets in 4 h of irradiation, which is about 6-fold higher than bare g-C_3_N_4_.

In the past few years, g-C_3_N_4_-based nanocomposites with semiconductors and metals have been successfully prepared and employed for environmental remediation applications for various harmful pollutant degradation. Some of the notable recent reports have been presented in Table 5.

**Table 5:** g-C_3_N_4_-based nanocomposites for environmental remediation. CQDs – carbon quantum dots; CNTs – carbon nanotubes; MO – methyl orange; MB – methylene blue; DCP – dichlorophenol; PNP – p-nitrophenol; RhB – rhodamine B; BF – fuchsin; 4-NP – 4-nitrophenol.

Sl no.	photocatalyst	light source	pollutant (concentration)	*t*_completion_ (min)	ref. (Year)

1	g-C_3_N_4_–Au	500 W Xe lamp	MO (10 mg L^−1^)	150 min	[223] (2013)
2	g-C_3_N_4_–Bi_2_WO_6_	300 W Xe lamp	MO (5 mg L^−1^), 2,4-DCP (20 mg L^−1^)	120 min	[224] (2013)
3	g-C_3_N_4_–Ag_2_O	300 W Xe lamp	MO, phenol (20 mg L^−1^)	30 min, 180 min	[225] (2013)
4	g-C_3_N_4_–Ag	300 W Xe lamp	MO, PNP (10 mg L^−1^)	120 min	[226] (2013)
5	g-C_3_N_4_–C–ZnO	300 W Xe lamp	MB (10 mg L^−1^)	120 min	[74] (2014)
6	g-C_3_N_4_–N–ZnO	300 W Xe lamp	RhB (5 mg L^−1^)	60 min	[227] (2014)
7	g-C_3_N_4_–ZnO	500 W Xe lamp	MB (0.04 mM)	150 min	[228] (2014)
8	g-C_3_N_4_–WO_3_	500 W Xe lamp	MB (0.9 × 10^−5^ mol), BF (1.0 × 10^−5^ mol)	60 min	[229] (2014)
9	g-C_3_N_4_–WO_3_	500 W Xe lamp	RhB (0.01 M)	120 min	[230] (2014)
10	g-C_3_N_4_–N–SrTiO_3_	300 W Xe lamp	RhB, 4-chlorophenol (5 mg L^−1^)	60 min	[231] (2014)
11	g-C_3_N_4_–CdS	PLS-SXE 300 lamp	MO (5 mg L^−1^)	16 min	[232] (2014)
12	g-C_3_N_4_–C_60_	500 W Xe lamp	RhB (1.0 × 10^−5^ mol L^−1^)	60 min	[233] (2014)
13	g-C_3_N_4_–C_60_	500 W Xe lamp	MB (0.01 mM), phenol (5 ppm)	180 min	[234] (2014)
14	g-C_3_N_4_–TiO_2_	100 W mercury lamp, 300 W halogen lamp	MO, RhB (0.2 wt %)	50 min, 300 min	[235] (2014)
15	g-C_3_N_4_–SnO_2_	300 W Xe lamp	MO (10 ppm)	180 min	[170] (2014)
16	g-C_3_N_4_–SnS_2_	300 W Xe lamp	Cr(VI) (50 mg L^−1^)	50 min	[236] (2014)
17	g-C_3_N_4_–Ag	500 W Xe lamp	MB (0.01 mM) and phenol (10 ppm)	–	[161] (2014)
18	C_3_N_4_–CQD	IR source	MO (4 mg L^−1^)	240 min	[237] (2015)
19	g-C_3_N_4_–Au–CNT	visible light source	RhB	50 min	[238] (2015)
20	g-C_3_N_4_ –TiO_2_	LED 3 W	MO, phenol (10 mg L^−1^)	80 min	[239] (2015)
21	g-C_3_N_4_ –Ti^3+^–TiO_2_	300 W Dy lamp	RhB (20 mg L^−1^)	120 min	[240] (2015)
22	g-C_3_N_4_–Ag_2_CO_3_	300 W Xe lamp	MO, RhB (10 mg L^−1^)	30 min	[241] (2015)
23	g-C_3_N_4_–AgBr	35 W metal halide lamp	MO (10 mg L^−1^)	120 min	[242] (2015)
24	g-C_3_N_4_–Bi_2_WO_6_	Xe lamp	RhB (10 mg L^−1^)	50 min	[243] (2015)
25	g-C_3_N_4_–CeO_2_	300 W Xe lamp	MB (10 mg L^−1^)	210 min	[244] (2015)
26	g-C_3_N_4_–Fe_2_O_3_	300 W Xe lamp	RhB (20 mg L^−1^)	90 min	[245] (2015)
27	g-C_3_N_4_–AgVO_3_	visible light	MO (10 mg L^−1^)	60 min	[246] (2017)
28	g-C_3_N_4_–Ag–Fe_3_O_4_	visible light	MB (10 ppm)	120 min	[247] (2017)
29	Na–g-C_3_N_4_–DyVO_4_	tungsten/halogen linear lamp (500 W)	RhB (0.02 mM), 4-NP (0.143 mM)	80 min, ≈360 min	[248] (2017)
30	g-C_3_N_4_–TiO_2_–CdS	500 W Xe lamp	phenol (10 mg L^−1^)	300 min	[249] (2017)

## Conclusion

The combination of excellent properties and the easy availability have made carbon-based materials one of the most promising materials for catalysis. Solar energy harvesting for energy generation from water is one of the attractive and challenging field in photocatalysis. Due to the huge specific surface area, graphene acts as an excellent 2D support material for metals, metal oxides and other materials. The tunable optical and electronic properties of these materials have made them a versatile material, particularly graphene, which can act as cocatalyst, photocatalyst and photosensitizer, and even exhibit the property of hydrogen evolution (energy generation) by itself. On similar note, a wide range of g-C_3_N_4_-based nanocomposites with non-metal, metal oxide semiconductors, composite oxide semiconductors, and noble metals have been reported with enhanced light absorption and accelerated charge transfer kinetics for energy generation applications. Furthermore, these two-dimensional carbon-based nanocomposites have shown promising results in the case of photocatalytic environmental applications as well, as described in detail in this review article.

Despite all the excellent results obtained with carbon-based nanocomposites for photocatalytic applications, there are also some challenges for improving its utilization.

(1) First of all, the water splitting reaction is a thermodynamically unfavourable reaction as the Gibbs free energy is positive for this reaction. Hence, making this reaction feasible and preventing the back reaction of hydrogen and oxygen to form water using economic and ecologically-friendly catalysts is a big challenge.

(2) The oxidation of graphite flakes introduces various functional groups in graphene oxide, which disrupt its electronic structure by several orders of magnitude as compared to pristine graphene. The conductivity is revived when graphene oxide is reduced but various defects remain. Thus, the fabrication of novel graphene-based nanocomposites with improved catalytic performance is still a challenge. Moreover, large-scale production of graphene-based nanocomposites with controlled morphology and high performance is a challenging task.

(3) The role of graphene as a photocatalyst and photosensitizer is also complex in a mechanistic way, because generally it has been reported that the enhanced photogenerated charge carrier separation, and then charge transfer to the CB of semiconductor, is responsible for the activity of the catalyst. However, many research groups have demonstrated that electrons can be transferred from the upper VB of graphene to the semiconductor, as graphene can act as photosensitizer. Such a mechanism is still not fully understood and detailed investigations are needed for this particularly interesting interfacial charge transfer in graphene-based nanocomposites.

(4) The multicomponent graphene-based nanocomposites have shown remarkable enhancement in the photocatalytic performance towards energy generation and pollutant removal due to improved charge transfer kinetics and well-defined intimate contact between constituent materials. Therefore, more of the facile synthetic strategies need to be developed in order to control morphology and design such multicomponent nanocomposites.

(5) In photocatalytic water splitting, it is predominantly the hydrogen evolution which contributes to the energy generation. This evolved hydrogen needs to be stored in an efficient and safe manner for future consumption. Hence, hydrogen storage is also a big issue in order to use it as fuel.

(6) Although a huge number of carbon-based photocatalysts have been explored for energy generation by solar water splitting, the significant breakthrough in harvesting energy by utilizing the full solar spectrum still needs to be achieved.

(7) Most of the photocatalytic water splitting reactions for H_2_ generation are carried out in the presence of sacrificial agents as hole scavengers, such as methanol, ethanol, triethanolamine, sodium sulfide, sodium sulphite, etc. Keeping in view the energy efficiency, environmental benignity and sustainability, the use of such sacrificial agents needs to be avoided in future.

(8) The synthesis of g-C_3_N_4_-based complex nanocomposites with proper architecture and a rational charge cascading process for real life applications is full of challenges as the mechanism of photocatalytic enhancement by g-C_3_N_4_ nanocomposites is still unclear.

(9) The most important concern with g-C_3_N_4_-based complex nanocomposites is stability, which is not well addressed to date. The photocatalytic stability is one of the crucial parameters that decides commercial application of catalysts.

(10) The detailed mechanistic pathways leading to the mineralization of pollutants using these carbon-based nanocomposites as photocatalysts is not fully understood and entails detailed investigations on the intermediates formed during the process.

Finally, the rapid development of materials science and nanotechnology in the past few years has invented a new class of functional materials for photocatalytic applications. The fascinating properties of these materials could be further explored for understanding the mechanisms in photocatalytic reactions to effectively address the various global issues in the future. Hence, it requires more effort from scientific community for better understanding of physicochemical properties of the nanocomposites based on these two-dimensional carbon-based materials to develop novel functional materials for sustainable chemistry.
